# SYNOSIS: Image Synthesis Pipeline for Machine Vision in Metal Surface Inspection

**DOI:** 10.3390/s25196016

**Published:** 2025-09-30

**Authors:** Juraj Fulir, Natascha Jeziorski, Lovro Bosnar, Hans Hagen, Claudia Redenbach, Tobias Herrfurth, Marcus Trost, Thomas Gischkat, Petra Gospodnetić

**Affiliations:** 1 Image Processing Department, Fraunhofer ITWM, 67663 Kaiserslautern, Germany; natascha.jeziorski@itwm.fraunhofer.de (N.J.); lovro.bosnar@itwm-extern.fraunhofer.de (L.B.); petra.gospodnetic@itwm.fraunhofer.de (P.G.); 2 Department of Computer Science, RPTU Kaiserslautern-Landau, 67663 Kaiserslautern, Germany; hagen@cs.uni-kl.de; 3 Department of Mathematics, RPTU Kaiserslautern-Landau, 67663 Kaiserslautern, Germany; redenbach@mathematik.uni-kl.de; 4 Fraunhofer IOF, 07745 Jena, Germany; tobias.herffurth@iof.fraunhofer.de (T.H.); marcus.trost@iof.fraunhofer.de (M.T.); thomas.gischkat@iof.fraunhofer.de (T.G.)

**Keywords:** synthetic data, defect recognition, surface texture, texture modeling, surface inspection, machine vision, domain generalization, data similarity, milling

## Abstract

The use of machine learning methods for the development of robust and flexible visual inspection systems has shown promising results. However, their performance is highly dependent on the large amount and diversity of training data, which is difficult to obtain in practice. Recent developments in synthetic dataset generation have seen increasing success in overcoming these problems. However, the prevailing work revolves around the usage of generative models, which suffer from data shortages, hallucinations, and provide limited support for unobserved edge-cases. In this work, we present the first synthetic data generation pipeline that is capable of generating large datasets of physically realistic textures exhibiting sophisticated structured patterns. Our framework is based on procedural texture modelling with interpretable parameters, uniquely allowing us to guarantee precise control over the texture parameters as we generate a high variety of observed and unobserved texture instances. We publish the dual dataset used in this paper, presenting models of sandblasting, parallel, and spiral milling textures, which are commonly present on manufactured metal products. To evaluate the dataset quality, we go beyond final model performance comparison by measuring different image similarities between the real and synthetic domains. This uncovered a trend, indicating these metrics could be used to predict downstream detection performance, which can strongly impact future developments of synthetic data.

## 1. Introduction

Automated machine vision has seen wide adoption in industrial applications to automate repetitive processes that rely on visual information. Visual quality inspection is a popular choice due to its versatility across different geometries and materials, cost-effectiveness, and inspection speed. It can be integrated in different stages of the manufacturing process, ensuring final quality, while minimizing the time and resource costs of inspecting an object. While manual visual inspection involving humans is a popular and often cost-effective choice for low-volume productions, an automated solution is more fitting for various scenarios, such as rapid production lines, continuous manufacturing, and extreme working environments (temperature, noise, chemicals, etc.). Although, machine vision systems are a flexible approach in terms of their variety of possible applications, they are highly specialized to the task at hand, designed using scarce expert knowledge and face adaptability issues under unforeseen inspection requirements or production environment changes.

Automated visual inspection systems (further referred to as inspection systems) are currently developed in a manner that requires a high level of rigidness to ensure efficient and reliable performance [1]. The most rigid component is the machine vision algorithm, which is commonly implemented using traditional techniques. While they produce interpretable results and can offer verification, they are specialized for a particular use case and are sensitive to changes in the environment. This, in turn, reduces the customization capabilities of production lines, slowing down innovation in manufacturing. To overcome this, the integrators of inspection systems are turning towards machine learning (ML) techniques for machine vision, which demonstrated higher flexibility to changes. However, their reliability and flexibility over longer periods of time is yet to be proven, making the industry skeptical towards their integration into production lines. Additionally, machine learning requires a significant amount of training data, which is a major challenge since the industry aims at reducing the frequency of defective parts, making such data scarce. The inspection tasks are typically very specific and, since there are little to no publicly available datasets [2], the task becomes time-consuming due to data collection.

To overcome these issues, practitioners are turning to synthetic data sources as a supplement to data shortages. In academia, the usage of synthetic data for ML is rapidly gaining popularity [3]. In industrial inspection systems, isolated studies have been performed providing evidence of its benefit. However, to date, there is a lack of commonly agreed upon best practices or a more comprehensive evaluation of the different steps that are required to generate a photorealistic synthetic dataset for visual inspection. Industrial inspection is facing scarcity of the datasets that are needed for benchmarking recognition models on surfaces with prominent microstructures, as well as dual datasets containing matching real and synthetic images for the development of domain alignment methods. To the best of the authors’ knowledge, no pipeline has been presented that can generate such data with a high level of realism and variety.

**Figure 1:**
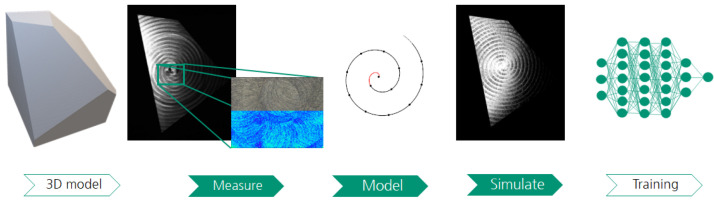
SYNOSIS synthesis pipeline. Based on a 3D model of an object, spatial properties of the surface texture are measured. Surface topography and manufacturing parameters are used to develop a mathematical model that is capable of reproducing the texture as a normal map. Multiple realizations of the texture are generated by varying the parameters and are applied onto the 3D model of the object that has been altered to include surface defects of varying types and sizes. During the simulation step, the final image is computed based on the interaction between the acquisition environment (light, camera), object geometry, and texture normal map. This process is repeated an arbitrary amount of times. The parameters that are defining the texture and surface defects are varied to generate a training dataset that is sufficient in terms of both image quantity and content variance.

**Figure 2:**
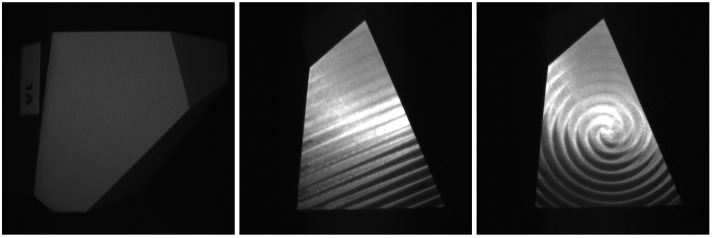
Examples of the three textures considered in this paper: surface finishing by sandblasting, parallel milling, and spiral milling.

As such, we introduce the SYNOSIS (synthetic, optically realistic images for ML-based inspection systems) pipeline (Figure 1). It is designed to fill the needs for generating realistic and controllable synthetic data that are specialized to a specific need, and it provides a backbone for the extension of support for other textured surfaces (e.g., casting, grinding, and additive manufacturing). Our contributions are as follows:

- This is a novel pipeline for designing a realistic, interpretable and controllable data generator, for the low-data regime of uncoated textured metal surfaces (Figure 2).
- The definition of the geometry scale decomposition is to include the notion of a surface’s microstructure in the synthetic object modeling nomenclature.
- The definition of dataset quality decomposition is to provide a more interpretable description of the domain gap and its source.
- Publication of a dual (real and synthetic) dataset that provides data for common processed metal surfaces, facilitates domain gap analysis, and serves the wider research community with a novel pattern recognition challenge.

### Paper Structure

The content is grouped into eight sections and contains bolded keywords to make the reading easier. The generation process that is needed to obtain synthetic images for surface inspection is covered in Section 2. There, we provide an overview of the related work, introduce the inspection-relevant decomposition of scales, and discuss the most important elements of the generation process and their relation to different scales. Section 3 and Section 4 introduce the methods chosen to produce test object surfaces in a controlled manner to measure surface topography. Section 5 and Section 6 focus on texture and defect modeling as the most relevant aspects that need to be controlled when creating synthetic data for surface inspection. Section 5 discusses the different approaches that can be taken to represent surface texture and their benefits or shortcomings. Further, the section introduces the stochastic geometry modeling approaches used for the specific surfaces found in our specific use-case. Section 6 discusses the different approaches used to defect generation in the related work, and provides an overview of the defect modeling workflow that is used for our specific use-case. Section 7 introduces how both the real and synthetic data were obtained. For synthetic data, it explains how the synthetic image generation process (introduced in Section 2) was used and with what parameters to create the synthetic dataset. Finally, Section 8 provides a discussion of the different means in which can be used to evaluate the quality of the created synthetic dataset, as well as the results when applied to our use-case.

## 2. Synthetic Dataset Generation

### 2.1. Related Work

The goal of synthetic image data generation is to generate image datasets where the image characteristics are ideally the same as those of images acquired in reality. The characteristics include both appearance and content distribution. When there is a discrepancy between the synthetic and real images in terms of their characteristics, it is referred to as the domain gap.

An early, straightforward, approach to extending training datasets was to add defect textures to the images of defect-free surfaces [4]. The defects are 2D image patches that do not correctly represent light interaction and have an inconsistent appearance with respect to the remainder of the surface. Due to the nature of the approach, it is very difficult to enhance it in a way that would minimize the resulting domain gap. Therefore, further synthetic data efforts were aimed at generative methods, based on predominantly deep learning, and rule-based approaches, using computer graphics simulation based on a well-defined set of rules that describe the imaging context [5].

The high degree of modeling complexity when generating photorealistic surface features caused many of the authors to adopt the generative approach, focusing on defect generation. Jain et al. [6] compare the performance of three different GAN architectures for generating defects in hot-rolled steel strips. Defect-GAN [7] was introduced for automated surface defect synthesis by mimicking the defacement and restoration process and capturing stochastic variation of defects. Defect-Transfer GAN [8] is trained over multiple products with similar defects in order to enable the introduction of semantically new information and, therefore, make up for the limited availability of all possible object attributes in the real dataset. Wei et al. [9] generated defect patches and blended them into a clean image, thus they were able to obtain approximate segmentation masks. These approaches are more suitable for generating large defects that visibly differ from the surrounding surface and do not suffer if an image is resized to a lower resolution for inference performance reasons. As such, they can be expected to struggle when confronted with a surface that has a texture pattern whose appearance has prominent local variations, as is the case for milled surfaces. Schmedemann [10] explored a hybrid approach by applying style-transfer on images generated by a rule-based approach to obtain more control and reduce the domain gap. It has shown that this approach can lead to a loss of features, making it unreliable without a human operator review of the generated images.

Generative deep learning approaches may result in images that look realistic but that also incorporate detail hallucinations or global structure errors, which can be difficult to detect by human eye [11,12,13]. These cause misalignments of the detector models and risk increasing the number of false negatives. Also, these methods offer limited reasoning over the parameters that generate a particular scene, severely limiting the precision control over them. Hence, it is difficult to control the dataset content and the distribution of features within, severely limiting the generation of the edge cases that are crucial for the reliable operation of inspection systems. In addition and in part due to data shortage, to date, no generative models capable of generating data for visual inspection exist up to the precise specification of the end product.

Rule-based image synthesis relies on computer graphics for modeling and rendering virtual environments, which have recently gained popularity beyond the entertainment industry (e.g., movies or gaming). It represents a controllable, versatile, and reliable approach for generating arbitrary amounts of data with customized features and variations. As such, it has proven useful across various machine vision tasks, as discussed by Dahmen et al. [14]. Synthetic data are currently predominantly used in human-oriented domains, autonomous driving, and robotics; see [15].

Human-oriented synthetic data are used in tasks that include body tracking and pose estimation [16,17,18] or face recognition [19]. Synthetic data for autonomous driving rely on virtual environments of large-scale urban and traffic scenes [20] for recognition tasks [21] and object detection [22]. The robotics domain requires virtual environments [23,24] for recognition tasks, such as semantic segmentation [25] and object detection [26]. In all three domains, the focus is on large scale geometric features while simulation of small scale details and textures is unnecessary.

Surface inspection belongs to industry automation, which is closely linked to robotics but includes tasks beyond those needed for movement, localization, and mapping. In particular, building virtual environments for inspection planning and the development of defect recognition algorithms require a high level of realism and small-scale surface details. This makes this domain highly different from the domains discussed above.

A general-purpose dataset generator was introduced by Greff et al. [27]. It promises realism (using physical-based ray tracing), scalability, and reproducibility, which are also covered by our pipeline. However, being a general framework, it requires a user to define a 3D scene for the particular task by using scripting. Assets for the 3D scene must be either created by the user or imported from general-purpose asset libraries. As such, data generation for specialized problems, such as quality assurance, is challenging due to unavailable assets, assets with unsatisfactory features, or limited modeling capabilities of a user.

Moonen et al. [28] presented a tool kit for synthetic image data generation in manufacturing. Synthesizing datasets for industrial inspection using a rule-based approach was further discussed in [29,30,31,32,33]. Synthetic data were further used for various quality assurance tasks, such as inspections of industrial components [34], metal surface inspections [35], industrial visual inspections [36], scaffolding quality inspections [37], or viewpoint estimation [38]. Raymond et al. [39] introduced models of scratched metal surfaces where they stacked layers containing different scratch distributions. However, their model does not represent curving scratches nor dents or scratches with varying geometrical depths. None of the mentioned works provide the level of control introduced by Bosnar et al. [40], with parameters designed to separately control image acquisition contexts, texture appearances, or defect characteristics.

The work presented in this paper builds on the virtual inspection planning research done by Gospodnetić [41] and Bosnar et al. [40,42,43]. Bosnar et al. [40] identified the core computer graphics components needed to perform image synthesis for surface inspection, and they stressed the importance of texture and defect parameterization to minimize the need for an artistic approach. However, it focused mainly on the concepts and not challenges that arise when applying it to a realistic scenario. We extend the aforementioned pipeline and apply it to a realistic scenario. Procedural textures and defects for generating synthetic data for defect recognition in visual surface inspection were introduced in [42,43]. Their defect modeling approach was also adopted by Schmedemann et al. [44] for the rule-based generated images before application of style transfer. Fulir et al. [45] also built on top of [40,42,43] to produce a synthetic dataset for defect segmentation on metal surfaces; however, they mainly discussed ML results.

### 2.2. Decomposition of Scales

A 3D scene contains 3D objects that are traditionally decoupled into geometry and material. The geometry specifies the size, position, and shape of an object (high-level appearance), while the material influences how the light interacts with its surface (detailed appearance). Alternatively, a 3D object can be considered at three different scales as follows: macro-, meso-, and microscale. **Macroscale** refers to object geometry (e.g., represented by a mesh) and large geometrical features such as geometrical defects. **Mesoscale** refers to the surface structure on a much smaller scale than the shape of the object but larger than the wavelength of light (i.e., surface texture), as discussed by Dorsey et al. [46] (ch. 2). **Microscale** refers to the surface structure and properties that are not separately distinguishable by the imaging sensor but are contributing to light reflection.

It is difficult to make a strict distinction between the scales using units because the scales are relative to the imaging sensor resolution and viewing distance. Therefore, we propose the following guidelines: The macroscale should be attributed to geometrical shapes that take larger portions of the image. The microscale can be determined using the Nyquist–Shannon sampling theorem [47], stating that the sampling rate must be at least twice the bandwidth of the signal. In other words, every feature that cannot be sampled by more than one pixel falls under the microscale class. Finally, the mesoscale constitutes features that occupy a smaller but visible portion of the image, which are measured in a handful of pixels.

To give some examples, assume that we were to observe an outdoor scene of a park containing people and trees. Then, the shapes of the people and trees would make the macroscale, whereas the eyes or leaves would already fall under the mesoscale, and the skin or the leaf veins would be considered microscale. In the case of visual surface inspection, we are looking at specific parts of an object with a high resolution. Therefore, the inspected object is in the macroscale, its defects and surface texture caused by the manufacturing process are in the mesoscale, while roughness and smallest surface features are part of the microscale.

### 2.3. Rule-Based Image Synthesis

**Figure 3:**
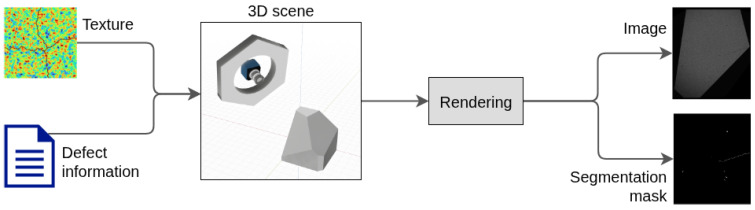
Image synthesis overview. The texture and defect information are joined with the 3D scene to perform rendering and to generate an image. The defect information is varied to perform photo-realistic image synthesis of both defected and defect-free object instances. In case of defected instances, pixel-precise defect annotations are automatically created.

Rule-based image synthesis (Figure 3) can be divided into two main steps as follows: 3D scene modeling (i.e., modeling of the virtual environment) and rendering procedures, as discussed by Greenberg et al. [48] and Tsirikoglou [15]. During rendering, the 3D scene is used to generate 2D images. The crucial element for achieving realism of those images is physical-based light transport, using path-tracing (Kajiya [49], Pharr et al. [50]). In this work, we used the Appleseed [51] rendering engine, which implements these principles.

### 2.4. 3D Scene Modeling

The 3D scene represents the real-world context within which the images are acquired. As such, the scene to be simulated must faithfully represent the environment. For surface inspection, this includes 3D objects, lights, and cameras. Note that we do not necessary include environment illumination since inspection systems often have a strictly controlled acquisition environment and no uncontrolled illumination. While all the components contribute to the image’s realism, in this work, we focus on the realism of a 3D object, influenced by the geometry, material, and surface topographies (i.e., texture).

The geometry of the 3D models representing the inspected object (macroscale) must correspond to the geometry of the real inspected product. In this work, we use the 3D models of the test bodies (Section 3) and create defected product instances by augmenting defects (mesoscale) directly into the product’s geometry [43]. This is done because the defects are larger than the surface texture, and their complex shape causes intricate light scattering that can not be captured by normal maps alone, as described in Section 6.

Material properties describe the light–surface interaction and are modelled using the bidirectional reflectance distribution function (BRDF), as discussed by Dorsey et al. [46]. For this work, rough metal surface BRDF is used (Walter et al. [52], Kulla et al. [53], and Turquin et al. [54]). The BRDF is evaluated in every surface point based on the local surface normal. Unrealistically smooth surfaces are avoided through variations in the BRDF parameters and normals using texture.

Texture is a term for which the exact definition differs across domains. Here, we refer to the computer graphics concept that defines it as a procedure to introduce the surface topography by influencing (perturbing) the surface normal and BRDF parameters for every surface point at the rendering time (Mikkelsen et al. [55]). As such, the texture describes the mesoscale surface patterns, which would otherwise be very inefficient if they were represented by the geometry. Section 5 offers a more complete overview of the texture modeling approaches. Traditionally, texture modeling is aimed to be used by artists to approximately recreate a photorealistic visual appearance and enable their creative expression (Guerrero et al. [56], Adobe Substance [57], Ebert et al. [58]). Therefore, the surface models are based more on artist-oriented parameters rather than real-world parameters, making them visually appealing but not necessarily correct. Dong et al. [59] approached realism by fitting a Gaussian random field to surface measurements and using it as a texture. It gave impressive results for a single instance of measurements, but offered no further control over the pattern’s properties.

In contrast to [59], we recreated the surface measurements by using mathematical models that can be controlled based on real-world parameters (see Section 5). The textures are generated as 2D images, encoding the normal variations, which are then mapped onto the surface of the object, as explained in Section 2.5. Given the high level of details produced by the models, we kept the BRDF roughness parameter fixed to constant small values (e.g., within a range (0.0–0.1)).

Light in the 3D scene represents the illumination devices used in the surface inspection environment. We used a model of the ring light geometry used in the real inspection system, which has a torus-like shape (Figure 3) with diffuse emission distribution describing the light emission in each point of the shape. As in the real setup, the camera was placed in the center of the ring, with both objects having the same orientation.

The camera used a pinhole camera model, defined by its resolution, pixel size, focal length, position, and orientation parameters. Simulated camera parameters are set to resemble the real camera parameters used in the surface inspection environment. The pinhole model does not account for the depth of field or optical aberrations, such as distortions. For the purpose of this work, this is sufficient since the inspected surface is expected to be in focus, and effects, such as image distortion or global blurring, can be introduced in post-processing or during training in the form of image augmentations.

### 2.5. Texture Mapping

The textures used in this work are represented as normal map images, where each pixel encodes a normal vector. While [60] suggests that the implicit procedural generation is ideal, it requires an additional level of restrictions and complexity when developing the new models. In particular, it is the limited spatial information that makes describing spatially varying patterns with regular global structures challenging. In this work, the milling patterns (Figure 2) pose a great challenge, and the sequential generation of rings on-the-fly would be intractable for implicit procedural generation. While the implicit implementation is possible, it is outside the scope of this work in order to allow us to focus on physically realistic texture modeling.

In order to use the texture images together with a BRDF evaluation during rendering, it is required to map them onto the 3D object. Typically, it is either done by using projection directly [50] (e.g., planar, cube, or spherical) or by unwrapping the object [61] (i.e., mapping the object faces to a plane). Applying the texture projection directly onto the whole object will cause artifacts, such as texture stretching, for complex geometries. This is not acceptable when generating synthetic training data. Therefore, in this work, we first separated the geometry into main planar surfaces, and then we used planar projection separately per surface. To avoid the occurrence of texture tiling (visible repetition in pattern), the texture images were generated large enough to cover the entire target surface.

### 2.6. Rendering

The 3D scene description was used to render the synthetic images from all given camera positions. The main rendering parameters influencing the quality of the synthesized images were the number of samples per pixel (SPP) and the number of light ray bounces.

In surface inspections, each pixel covers rich and complex surface structures. A hHigher SPP ensures that the computed pixel color is a good approximation of the mean light response value of the covered area. As such, increasing the number of SPP will reduce the noise and aliasing in the rendered image.

In addition to texture, our 3D objects contained defects in the form of small, complex, geometrical features. Increasing the number of light bounces (i.e., the times that the ray is allowed to bounce off the intersection point) enables us to represent complex light phenomena, such as shadowing, masking, or interreflections. These phenomena can not be correctly represented using normal maps alone.

### 2.7. Defect Annotations Generation

**Figure 4:**
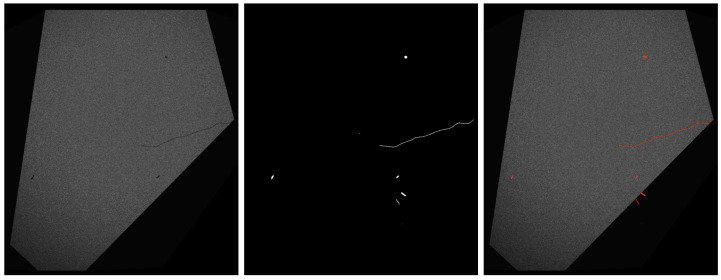
**Left**: defected synthetic image. **Middle**: defect annotations. **Right**: defected synthetic image with annotations as a red overlay.

The object geometry used in the 3D scene is defected using methods based on work by Bosnar et al. [43]. Each imprinted defect geometry has a corresponding geometrical defect mask; see Section 6. Black (non-emissive) material is applied on the 3D object, emissive material is set to the geometrical masks, and all light sources are disabled. Finally, the annotations are generated using the same camera parameters as for the corresponding photorealistic image, which ensures that the pixel-precise labels of the defect areas are visible from the camera position and not occluded by the 3D object. This approach was introduced by Bosnar et al. [43] and will always generate defect annotations, regardless of their light responses, as can be seen in Figure 4.

## 3. Test Body Design

Injection of the domain knowledge is crucial for developing photorealistic models. Therefore, we used custom designed and manufactured test objects, giving us complete insight into and control over the processing chain. This allowed us to focus on the image synthesis, surface modeling, dataset generation, and training challenges.

In total, three identical sets of test objects were fabricated, each containing 10 polyhedral test bodies. When designing the test bodies, the aim was to generate bodies with multiple faces that showed different realistic surface texture patterns.

**Figure 5:**
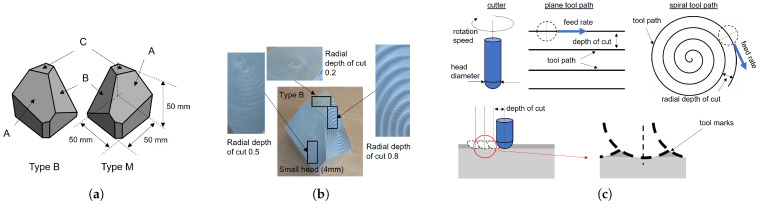
Illustration of the test object used in the project, the milling processes, and a test object with milled surfaces. (**a**) Drawing of the base test bodies. (**b**) Example picture of a type B test body with spiral milled surfaces. (**c**) Sketch of the milling processes and its main parameters.

The base bodies (type B and type M) are polyhedrons that are made from aluminum; see Figure 5a. Each polyhedron has a size of 5 cm × 5 cm × 5 cm with the planar faces A, B, and C having approximate sizes of 2 cm × 2 cm, which are measured and modeled subsequently. The remaining faces are left “unfinished” (rough milling) and are not further considered. The difference between the type B and type M is the mirrored arrangement of the faces of the base’s body.

First, the surfaces were milled using different processing parameters (Figure 5c). A rotating cutter with a specific head diameter was used to remove material from the surfaces by performing many separate small cuts along the tool path with a defined feed rate. We considered face-milling, which means that the tool moves perpendicularly to the object’s surface. The distance between the neighboring tool paths depends on the radial depth of the cut and influences the resulting tool marks; see Figure 5b. Furthermore, parallel linear and spiral tool paths were used. Artifacts in the form of exit lines can occur with spiral milling.

After milling, some of the faces were sandblasted—a process in which a hand-held nozzle of a sandblasting tool blasts the surface with a mixture of sand and air at a high pressure. This abrasive method can smoothen or roughen the surface. The resulting surface topography depends, thus, on the pressure of the air jet, the grain size distribution, and the shape and material of the sand. The parameter values of both processing techniques (see Table 1) were chosen so as to obtain the realistic patterns that were expected to emerge from industrial processing.

**Figure 6:**
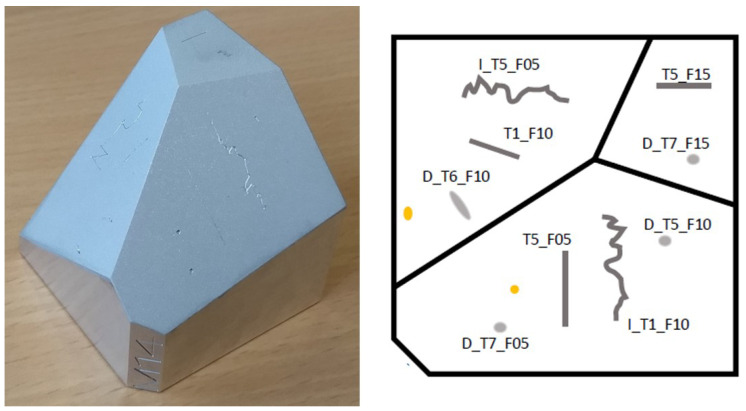
Test object type M after sandblasting and the subsequently introduced defects with different types and sizes.

**Figure 7:**
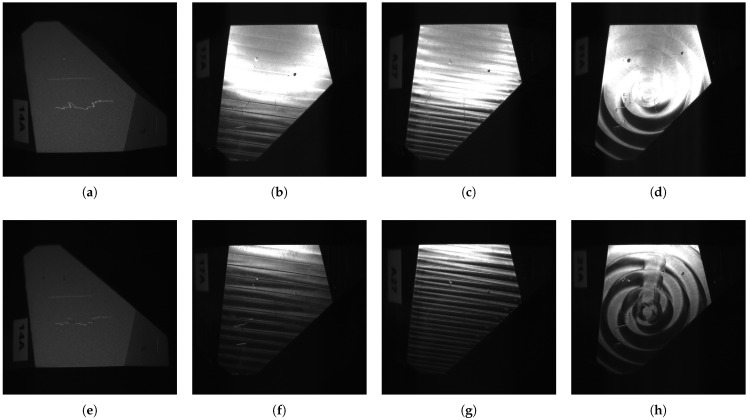
Real images of the object’s surface with defects. Top row: 20-degree angle from the perpendicular view. Bottom row: 40-degree angle from the perpendicular view. (**a**) Test body 14, face A. (**b**) Test body 17, face A. (**c**) Test body 27, face A. (**d**) Test body 21, face A. (**e**) Test body 14, face A. (**f**) Test body 17, face A. (**g**) Test body 27, face A. (**h**) Test body 21, face A.

Objects in set 1 contain no surface defects, while sets 2 and 3 contain more than 200 scratches and localized point defects, created precisely on the faces. For this purpose, a custom “indenter” and a scratch test tool were used, where the tips can be loaded with different masses. For the tips, we used a diamond tip, a steel needle, and a screw. The loads of the tips were 500 g, 1000 g, and 1500 g. Using the tools, the scratches (straight and free-form lines) and digs (dents) were realized on the surfaces as typical surface defects in accordance with ISO8785 [62]. The generated defect sizes were in the range of sub-millimeters to millimeters. See Figure 6 for an example of the defect specifications and Figure 7 for the examples of the imaged test bodies’ appearances.

## 4. Material Measurements

In order to characterize the machined surfaces, optical 3D and topography measurements were performed by means of focus-variation microscopy (Bruker alicona InfiniteFocus G4, Bruker, Billerica, MA, USA). In the high precision machining industry, measurement systems based on the focus-variation technique are standard inspection tools to characterize 3D mechanical parts. The advantage is the high precision and contact-free measurement of roughness, surface structure, micro-geometry, and form by using one optical sensor. The measurement system is based on a precise optical lens system with shallow depth sharpness. By changing the working distance between the measured surface and the microscope lens, different depths of the surface come into focus and are projected sharply onto the sensor. The proprietary software analyzes the distance for each point and measures the sharpness, which is used for the calculation of the surface depth profile (topography).

**Figure 8:**
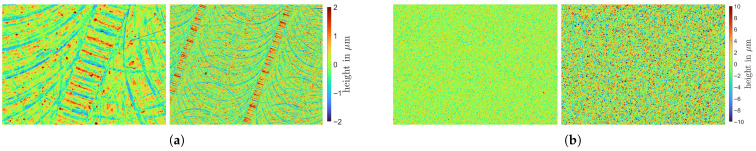
Topography measurements of sandblasted and milled surfaces. (**a**) Parallel milled surface using a milling head diameter of 4 mm and a radial depth of cut of 0.5. Small imaged region (**left**) is 2mm×1.4mm with a pixel size of 0.44 µm, and the large imaged region (**right**) is 7.5mm×5.9mm with a pixel size of 1.75 µm. (**b**) Sandblasted surface using pressure of 2.5 bar (**left**) and 6 bar (**right**). Imaged region is 7.7mm×5.7mm with a pixel size of 1.75 µm.

Depending on the magnification of the optical lens system, this process allows a vertical resolution down to 10 nm (a magnification equivalent to 100×, which is a physical magnification limit). For the purpose of this work, an optical lens system with a magnification of 5× and a nominal vertical resolution of 410 nm was used. The vertical resolution was limited during data acquisition by the measurement software. The defect-free samples of objects in test set 1 were measured with different lateral resolutions, depending on the size of the scanned surface area, as it is shown in Figure 8. The topography measurements are converted into ASCII xyz-files in order to create readable files used for surface modeling. This results in height images, where each pixel is assigned a height value.

**Figure 9:**
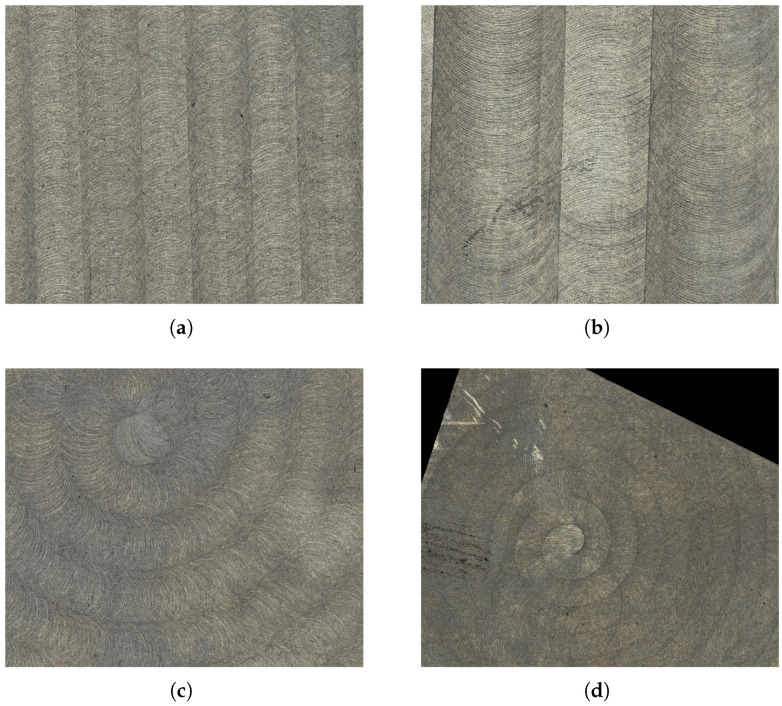
Optical 3D measurements of the milled surfaces using different parameter settings. Imaged region is 21mm×17.5mm with a pixel size of 7 µm. Best viewed digitally. (**a**) Parallel milling: a head diameter of 4 mm and a radial depth of cut of 0.8. (**b**) Parallel milling: a head diameter of 8 mm and a radial depth of cut of 0.8. (**c**) Spiral milling: a head diameter of 4 mm and a radial depth of cut of 0.5. (**d**) Spiral milling: a head diameter of 8 mm and a radial depth of cut of 0.2.

Depending on the process parameters, the tool marks created by the milling process form a unique pattern that can be observed by the naked eye (Figure 5b) and influences the subsequent surface light response, playing a key role in surface inspection. Measurements of the surfaces that were processed using different parameter settings are given in Figure 9.

When sandblasting, the previous milling does not affect the final surface pattern; see Figure 8b. The process results in a homogeneous surface texture without long scale spatial relations.

**Figure 10:**
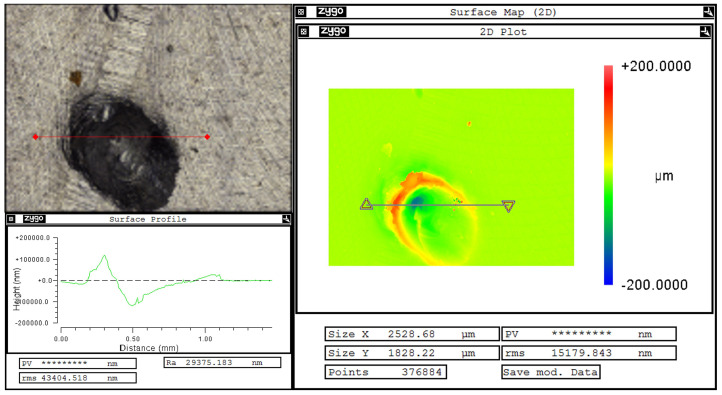
Software GUI with the optical 3D (**top left**) and topography (**right**) measurement with 2D intersection of the height profile (**bottom left**), which is marked in the topography measurement of a defect created using the indentor with a load of 500 g.

In order to characterize the generated defects, we used an optical lens system with a magnification of 20× and manually adjusted the vertical resolution during data acquisition through the software. The depth quality filter value was set between 2e-6 and 8e-6, depending on the defect type and size (e.g., Figure 10). The shape of the dent resembles a dig with a peak and a valley of about 150 µm in height and depth, with respect to the undamaged surface level.

## 5. Texture Modeling

Due to the very different surface topographies (Figure 8), separate models are required for sandblasted and milled surfaces. Models are selected based on the corresponding topography measurements M, and used to simulate the mesoscale of the object (texture). For completeness, in this section, we provide an overview and description for the process of modeling a procedural texture for the aforementioned surface microstructures, relying on the detailed process description presented by Jeziorski et. al. [63].

Measurements of the test body surfaces are provided as 2D height images $M:\left\{1,\ldots ,M_{M}\right\}\times \left\{1,\ldots ,N_{M}\right\}\to R$ of size $M_{M}\times N_{M}$ for $M_{M},N_{M}\in N$ and pixel spacing $\nu_{M}\in R_{>0}$. The output of the models is a texture image T of arbitrary size $M_{T}\times N_{T}$ and pixel spacing $\nu_{T}\geq \nu_{M}$ coarser or equal to the input’s pixel spacing. For the purpose of this work, we use $\nu_{T}\approx 6.1$ μm and $M_{T}=N_{T}=13,107$ so that a squared imaged region with an edge length 80 mm is provided. The chosen pixel spacing is large enough to generate images of appropriate size for which the rendering process can still be done in acceptable time. On the other hand, pixel spacing is chosen small enough so that no visible discretization artifacts occur. The resulting texture image can completely cover the test body surfaces during the rendering process, even when the texture image is rotated. This way, image tiling is avoided, as it may produce visible edges between the tiles.

### 5.1. Related Work

**Figure 11:**
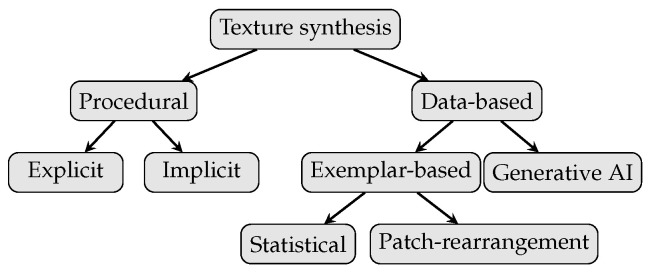
Classification of computer graphics methods for texture synthesis.

In computer graphics, the textures representing surface topographies can be roughly separated into procedural and data-based, see Figure 11. In this work we used explicit procedural for the milling texture and an exemplar-based approach for the sandblasted texture.

Procedural texture modeling is based on algorithms that completely describe the surface patterns without the need for additional data (e.g., image data). The main concept is to combine regular patterns (e.g., sine functions, simple shapes such as lines and circles, as well as other mathematical functions) discussed by Vivo et al. [64] with irregular patterns (e.g., noise functions), as discussed by Dong et al. [65]. Since procedural texture models are algorithmic descriptions of a texture, they offer parameterization and, thus, immense controllability, which is recognized by the computer graphics community for content creation [66]. However, developing procedural textures is often done by artists who base their work on experience and expression rather than encoding physically correct parameters.

Deguy [66] separates procedural models into implicit and explicit. An explicit procedural texture model generates a texture image that is first mapped onto the object’s surface and then used during rendering. Contrary, an implicit procedural texture model is directly evaluated during the rendering procedure, thus generating texture in render-time and allowing render-time re-parametrization. While explicit procedural models are easier to develop for complex texture patterns, implicit procedural texture models can cover arbitrarily large surfaces without repetitions or seams and are not restricted by the resolution. Bosnar et al. [42] developed implicit procedural texture models for circular, parallel, and radial textures. Those patterns resemble processed surfaces but without taking an explicit machining method into account or basing them on real surface topography.

Data-based texture modeling relies on sensor data and measurements as input, as discussed by Tsirikoglou et al. [15]. For example, the input texture image may be obtained using photogrammetry [67] or other scanning techniques [68].

The advantage of data-driven texture modeling is that it can synthesize approximations of real-world surfaces for which there are no well-defined mathematical descriptions. However, those methods offer little control over the generated content since they depend entirely on the input data. Data-based texture modeling can be roughly separated into generative AI and exemplar-based approaches. Generative AI texture synthesis is often based on GANs, as discussed by Jetchev et al. [69], Bergmann et al. [70], and Zeltner et al. [71]. While the results of these models often have remarkable appearance to a person, it is not possible to ensure that the results are physically correct, and it is very difficult to introduce controllable parameters for different types of textures. Exemplar-based texture synthesis uses algorithms to generate new texture images similar to a given input image. While stationary textures can be well reproduced, problems occur for textures with complex, large structures, e.g., milling rings.

Furthermore, since the output image relies entirely on the input image, it leaves no possibility of influencing the pattern itself. Raad et al. [72] gave an overview of the common exemplar-based texture synthesis methods. They distinguished between statistical and patch-rearrangement methods.

Statistical exemplar-based methods perform two steps. First, model-dependent statistics of the input image are computed. Second, a randomly initialized image is adjusted such that it fits the observed statistics. The asymptotic discrete spot noise (ADSN) and the random phase noise (RPN) are both parameter-free and non-iterative statistical methods [73]. A texture image simulated by the ADSN maintains the mean of the input image but has minor differences when comparing the sample auto-correlation since its Fourier modulus is the Fourier modulus of the input image multiplied by Rayleigh noise. The RPN creates texture images that have the same Fourier modulus as the input image and, thus, the same auto-correlation but with a random Fourier phase. Heeger and Bergen [74,75] introduced a procedure for texture modeling that uses image pyramids. A random image is adjusted iteratively by matching the histograms of all images in its image pyramid to the corresponding images in the image pyramid of the reference image. Portilla and Simoncelli [76,77] improved this method by using statistics such as cross-correlations between pyramid levels instead of histogram matching. In contrast to the exemplar-based methods that generate completely new texture images, the patch-rearrangement methods quilt patches taken from the input image. With the method of Efros and Freeman [78,79], the output image grows successively by adding certain patches one after the other in a raster-scan order. It is an extension of the method of Efros and Leung [80], which generates a new texture image pixel by pixel.

Guehl et al. [81] introduced a hybrid approach combining procedural and data-based texture synthesis. The visual structure of the generated image texture is based on a procedural parametric model, while texture details are synthesized using a data-driven approach. Another hybrid approach is presented by Hu et al. [82], in which the texture of the input image, which contains the BRDF parameters, is decomposed and proceduralized.

Modeling of metallic surfaces in computer graphics has received a lot of attention. Particularly interesting methods are the ones describing highly specular surfaces with high-frequency details (glints), as discussed by Zhu et al. [83] and Chermain [84]. Glint effects appear due to complex and unstructured small-scale, high-frequency geometries, such as tiny bumps, dents, and scratches. The problem with these approaches is that they do not model the actual properties of surfaces to resemble any particular and standardized surface. Rather, they are modeled to resemble the surface finishing appearance in an artistic way that is difficult to compare to any particular real-world counterpart.

Milling belongs to the machining processes resulting in deterministic and describable surface patterns [85,86]. Therefore, it is extremely useful to incorporate existing domain knowledge into texture models for milled surfaces. To estimate the surface quality of milled surfaces and, thus, determine the required quality, Felho et al. [87,88] and Kundrak et al. [89] developed models for parallel face-milled surfaces. Hadad et al. [90] developed a more detailed model that takes other typical milling parameters into account, e.g., tilting of the tool to avoid re-cutting. All models create milled surfaces using CAD software, and the patterns are, thus, geometrically imprinted into the surface. Oranli et al. [91] provided a similar approach to simulate the physical process of sandblasting and the resulting surface deformation in high precision using the Abaqus/Explicit 2019 software. Using such an approach in data synthesis would result in unfeasibly long computation times. Furthermore, the surfaces generated in this way look too perfect, as possible irregularities, e.g., slight deviations in the cutting geometry due to irregular material and machine behavior, are not included in the models.

### 5.2. Sandblasted Surface

The surface topography shows a different range of height values and degrees of roughness, depending on the pressure of the air jet (Figure 8b). The resulting surface topography is homogeneous, resembling a stationary Gaussian random field. However, fitting parametric Gaussian random fields did not provide satisfactory texture images since the structure sizes were not reproduced correctly. In addition, due to the small number of process parameters, it is difficult to develop a model that depends exclusively on them. Therefore, we used a combination of exemplar-based texture synthesis methods that only receive the measurements as input. We selected measurements with sizes between 4430×3248 and 4440×3288 and pixel spacing $\nu_{M}\approx 1.75$ µm that correspond to an area of about 7.8mm×5.8mm.

To generate texture images for sandblasted surfaces, the asymptotic discrete spot noise (ADSN) introduced by Galerne et al. [73] was used. The size and pixel spacing of the input image were maintained. To obtain a new texture image by simulating the ADSN, a sample from the multivariate normal distribution $N({\hat{\mu }}_{M},{\hat{C}}_{M})$ is taken, with ${\hat{\mu }}_{M}$ being the sample mean and ${\hat{C}}_{M}$ the sample covariance matrix of the input image.

**Figure 12:**
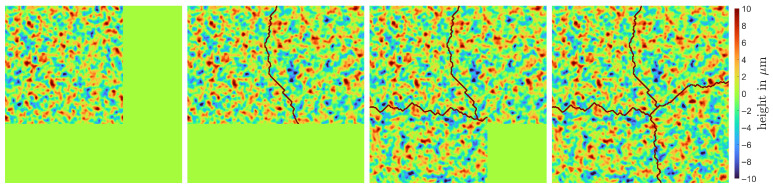
Illustration of EF stitching marking the minimal path in each step. Patches have a size of 512×512, and the overlap region width is 256 pixels. The imaged region is approximately 1.34mm×1.34mm.

Next, the desired size $M_{T}\times N_{T}$ and pixel spacing $\nu_{T}$ of the texture image have to be met. The pixel spacing can be adjusted by downsampling the input image using nearest neighbor interpolation. ADSN can generate texture images larger than the input image while maintaining the same pixel spacing [73]. Therefore, the normalized input image $\sqrt{M_{T}N_{T}\;/\;M_{M}N_{M}}\left(M-{\hat{\mu }}_{M}\right)+{\hat{\mu }}_{M}$ is enlarged using padding with mean value ${\hat{\mu }}_{M}$ before applying the ADSN. However, periodical repetitions are visible in the resulting texture. To avoid this, multiple texture image patches are generated and stitched using EF-stitching [78,79]. To stitch two neighboring patches within an overlap region, both are cut apart along an intersection edge first and put together afterward. The intersection edge is determined by finding the path within the overlap region where both images are most similar. Depending on the alignment of two neighboring patches, we distinguished between vertical, horizontal, and L-shaped stitching (Figure 12); see [63] for details.

### 5.3. Milled Surface

**Figure 13:**
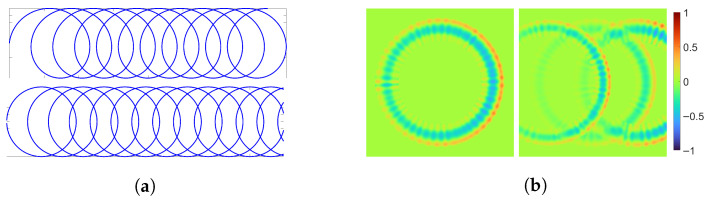
Overview of model generating milled surfaces. (**a**) Edge path (**top**) and its approximation (**bottom**). (**b**) Sub-models for one ring (**left**) and interaction of multiple rings (**right**).

The model generating textures of milled surfaces is given as a function in a continuous domain $R^{2}$. Thus, it is procedural but implemented explicitly for the sake of simplicity. The discrete texture image is obtained by evaluating the function at the image grid points $\left(\nu_{T}x,\nu_{T}y\right)$ for $x=1,\ldots ,M_{T}$ and $y=1,\ldots ,N_{T}$. The milling pattern appearance depends on a number of parameters associated with the production parameters. The most prominent structures are the cycloidal edge paths caused by the rotation of the milling head. They are approximated by the n∈N rings $R_{1},\ldots ,R_{n}$ (Figure 13a). The model is further divided into the following sub-models for the:

1. Tool path providing the arrangement and order of the rings by defining their center points $c_{k}$;
2. Appearance of an individual ring $R_{k}$ (Figure 13b);
3. Interaction between neighboring rings (Figure 13b).

For k=1,…,n. The heatmap scale is omitted, as the method is agnostic to the vertical scale.

**Figure 14:**
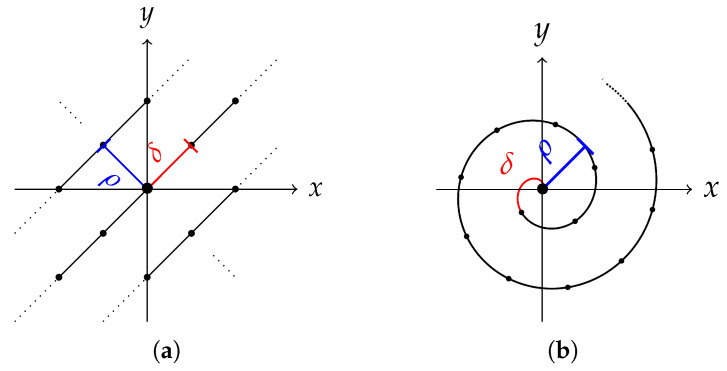
Tool paths with center points. (**a**) Parallel milling. (**b**) Spiral milling.

The first sub-model generates the ring center points $c_{k}\in R^{2}$ along the tool path. Within this study, either parallel or spiral milling (Figure 14) is considered. First, the respective tool path has to be modeled, together with the distance ρ∈(0,d) between two neighboring sub paths. The distance is controlled by the amount of ring overlap α∈(0,1) and the diameter $d\in R_{>0}$ of the rings, as ρ=(1−α)·d. The diameter of the milling head is crucial for defining the ring diameter, and the ring overlap depends on the radial cutting depth $a_{e}$ by $\alpha =1-a_{e}$. The radial cutting depth defines the distance that the tool steps over in relation to the ring diameter. As the blades do not reach the edge of the tool, α is reduced here by taking α−γ with γ∈[0,α). The ring center points $c_{k}$ are then modeled by random displacements of points ${\tilde{c}}_{k}$ that are computed along the tool path. That is, we sample from a two-dimensional normal distribution $c_{k}∼N\left({\tilde{c}}_{k},\Sigma_{c}\right)$. The location of ${\tilde{c}}_{k}$ is specified by the distance $\delta \in R_{>0}$ between two center points along the tool path. The distance δ depends on the tool’s feed rate and rotational speed. It is important to keep the chronological order of the rings in order to keep the tool path visible. Finally, while being very precise, the milling process is not perfect, and every once in a while, there is an occurrence of a ring that is more visible than the others. To introduce such irregularities in visibility of rings, the order of a certain amount ϵ∈[0,1] of rings is changed.

**Figure 15:**
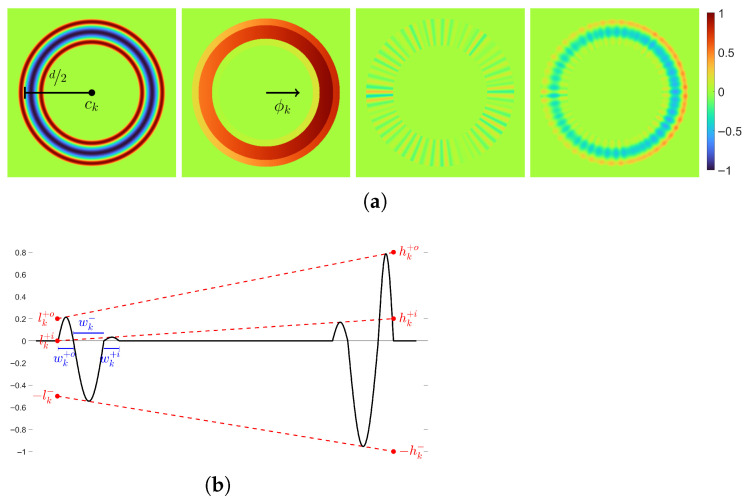
Illustration of sub-model for ring appearance with explanation of its parameters. (**a**) Sub-model for ring appearance using $\phi_{k}=0$, from left to right: $S_{k}$, $T_{k}$, $N_{k}$, and $R_{k}$. (**b**) The 1D intersection of the ring displaying the tilting mechanism: $S_{k}\;\cdot \;T_{k}$.

The second sub-model controls the appearance of an individual ring $R_{k}$, produced by a single rotation of the milling head cutting edge. Therefore, the width of the cutting edge is crucial for the indentation width $w_{k}^{-}∼N\left(\mu_{w^{-}},\sigma_{w^{-}}\right)$. To obtain the height values, the shape of the indentation is modeled by the cosine function −cos(x) for x∈[−π/2,π/2] scaled to the width of the ring. Material depositions are modeled by accumulations beside the indentation in the form of the rings having positive values. The width for the inner and outer rings are $w_{k}^{+i}∼N\left(\mu_{w^{+i}},\sigma_{w^{+i}}\right)$ and $w_{k}^{+o}∼N\left(\mu_{w^{+o}},\sigma_{w^{+o}}\right)$, and the height component is, again, modeled using the cosine function. All functions are combined to form the shape $S_{k}$ of ring $R_{k}$ (Figure 15).

Thus far, perpendicularity between the milling head and the surface has been assumed. However, the milling head is often tilted forwards in direction of the tool movement to prevent re-cutting the surface. This direction is given by the angle $\phi_{k}\in \left(-\pi ,\pi \right]$ in the *x*-*y*-plane between the tool path and the *x*-axis at point ${\tilde{c}}_{k}$. Tilting introduces a slope within the rings defined by the minimal $l_{k}^{•}∼N\left(\mu_{l^{•}},\sigma_{l^{•}}\right)$ and maximal $h_{k}^{•}∼N\left(\mu_{h^{•}},\sigma_{h^{•}}\right)$ indentations and inner and outer accumulations at the ring’s front, respectively, back for $•\in \left\{-,+i,+o\right\}$ (Figure 15). Since the tool’s presence prevents pushing material to the inside, $l^{+i}$ and $h^{+i}$ are chosen small. Tilting $T_{k}$ is then applied by multiplying with the shape $S_{k}$.

Finally, noise $N_{k}$ is added, simulating the irregularities caused by resistances in the material or vibrations during the process. It is modeled by a combination of $\lambda_{k}∼P\left(\lambda \right)$ sine curves with varying frequencies $\tau_{k_{j}}∼P\left(\tau \right)$ and random shifts $\xi_{k_{j}}∼U\left(-\pi ,\pi \right)$ for $j=1,\ldots ,\lambda_{k}$. Here, P denotes a Poisson and U a uniform distribution. Thus, $R_{k}=S_{k}\;\cdot \;T_{k}+N_{k}$ defines the sub-model for the appearance of one individual ring (Figure 15).

**Figure 16:**
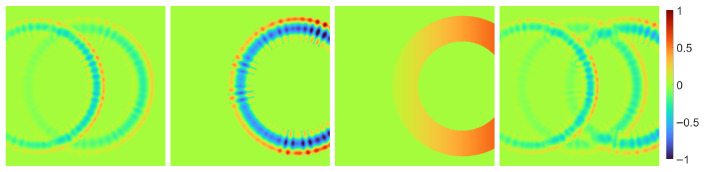
Sub-model for rings’ interactions using $\phi_{k}=0$ (no tilting), from left to right: $f(R_{1},R_{2})$, $R_{3}$, $L_{3}$, $f(R_{1},R_{2},R_{3})$.

The third sub-model deals with the interaction of the neighboring rings. First, the rings are successively added into the model, according to their order by using a recursive function $f\left(R_{1},\ldots ,R_{k}\right)=L_{k}\;\cdot \;R_{k}+\left(1-L_{k}\right)\;\cdot \;f\left(R_{1},\ldots ,R_{k-1}\right)$ for k=1,…,n and $R_{0}=0$ (Figure 16). Since every part of the surface is processed several times, all associated rings must be taken into account, with most of the weight on the temporally last ones. This is achieved by a convex combination. Weighting parameters are chosen as the linear function $L_{k}:R^{2}\to \left[0,1\right]$, which gives more weight to the front part of the ring than to the back. It is zero for points outside the ring. Values at the front and back of the rings are given by $a_{k}∼U\left(a^{\min},a^{\max}\right)$ and $b_{k}∼U\left(b^{\min},b^{\max}\right)$.

**Figure 17:**
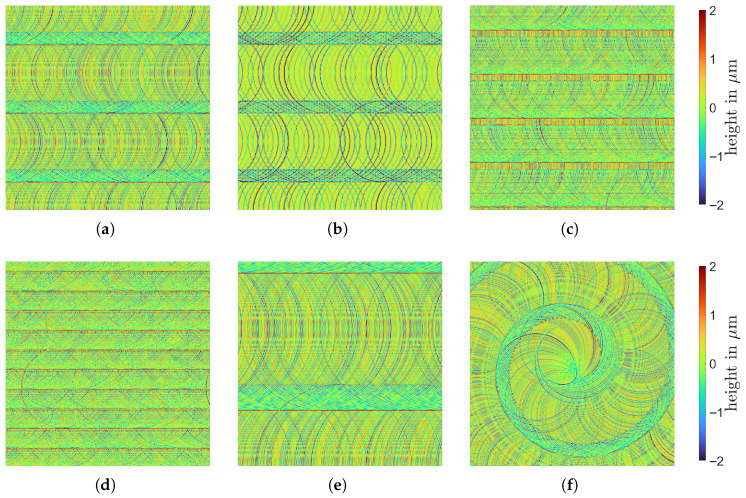
Adapted simulated milled surfaces generated with different parameter configurations. Imaged region is 10 mm×10 mm. (**a**) Parallel milling using d=4 mm, α=0.2, and δ=0.09 mm. (**b**) Parallel milling using d=4 mm, α=0.2, and δ=0.25 mm. (**c**) Parallel milling using d=4 mm, α=0.5, and δ=0.09 mm. (**d**) Parallel milling using d=4 mm, α=0.8, and δ=0.09 mm. (**e**) Parallel milling using d=8 mm, α=0.2, and δ=0.09 mm. (**f**) Spiral milling using d=4 mm, α=0.2, and δ=0.09 mm.

We refer to [63] for more details on the sub-models. A summary of the model parameters is given in Table 2. Some parameters are known explicitly (Table 1), while the others are estimated by visual comparison using high resolution topography measurements (Figure 8a). Simulations of the model using distinct parameter configurations are shown in Figure 17. Height values of the preliminary texture image $T_{\mathrm{pre}}$ are adapted so that their distribution resembles that of the corresponding measurement M. Mean values ${\hat{\mu }}_{•}$ and sample variances ${\hat{\sigma }}_{•}^{2}$, $•\in \{M,T_{\mathrm{pre}}\}$ are fit by setting $T=\sqrt{{\hat{\sigma }}_{M}\;/\;{\hat{\sigma }}_{T_{\mathrm{pre}}}}\;\cdot \;\left(T_{\mathrm{pre}}-{\hat{\mu }}_{M}\right)+{\hat{\mu }}_{T_{\mathrm{pre}}}$.

## 6. Defect Modeling

In this work, we used the approach of Bosnar et al. [43] to generate multiple instances of defected object geometry, overview of which is provided in this chapter.

The defected object geometry is created through procedural geometry modeling of dents and scratches, thus ensuring correct light scattering. The three main steps are (1) generation of defect positions, (2) generation of 3D geometries representing the defecting tools, and (3) imprinting the defecting tools into the surface and creating geometrical masks, which are used for generating defect annotations. Defect positions are determined by sampling points from an arbitrary distribution on the inspected product geometry. In this work, we used normal distribution. The defects are embedded into the geometry using defecting tools, i.e., negatives of dents (denting tools) and scratches (scratch tools). Denting tools are typically spherical and parameterized for controlling their scale, rotation, and shape. Scratch tools are created by performing a random walk on the product geometry, resulting in a path that is further converted into a solid geometry. The parameters are the thickness and curviness, which control the size and shape of the scratches.

The defecting tools are imprinted into the inspected product mesh by using a Boolean difference operation. Geometrical masks, used for annotation generation (see Section 2.7), are then created by using the inspected product mesh and the defecting tool. First, an intersection between the product mesh and the defecting tool mesh is computed. The resulting mesh represents a solid filling of the defect. The solid filling might be useful when the annotations must label missing areas of the object. In this work, we labeled only the visible defected surface and, therefore, performed an additional step that results in a mask shell. The defecting tool was first scaled with a factor in the range of [0.9, 1.0] and then it was intersected with the solid from the previous step.

**Figure 18:**
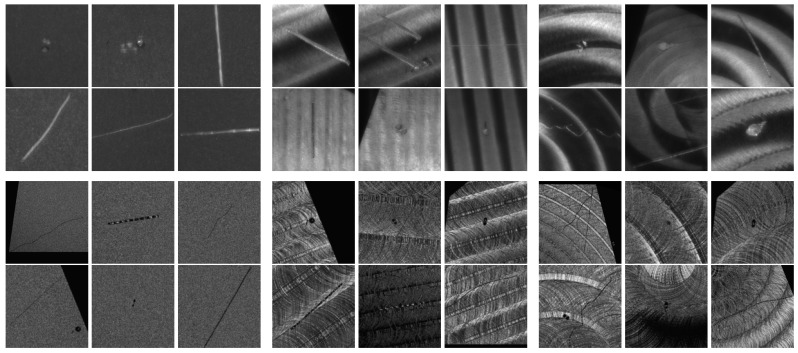
Real (**top**) and synthetic (**bottom**) crops of defects on different surfaces: sandblasted (**left**), parallel milling (**middle**), and spiral milling (**right**). The synthetic images are displayed before the application of pre-processing.

The defect parameters can be chosen to match the shape, size, and positions of the real defects observed in the measured defect topographies. However, more importantly, the parameter range can also be extended to produce defects beyond those observed in the available samples, i.e., rare occurrences or edge-cases. This enables the creation of an arbitrary number of defected 3D objects containing a wide variety of possible defects (see Figure 18) for which pixel-precise annotations are generated.

## 7. Dual Dataset

Publicly available datasets for inspection of metal objects mostly focus on defect recognition over flat surfaces and single-view inspection setups [92]. With it, they reduce the expected appearance diversity of defects and surfaces in the image, making the defect recognition task easier. However, such a restriction is not possible for an inspection of more complex objects where multi-view imaging is required and the visibility of the defects varies between different views. The aforementioned datasets contain objects, such as hot-rolled steel [93,94,95,96], slightly curved metal pads [97,98], rails [99], and pipes [100]. Datasets of smaller metal objects with complex geometry are also present; however, they are also under single-view inspection setups [101,102]. Multi-view inspection setups inspect the same surface multiple times while varying, at least, the direction of the incoming light source or the direction of the camera so as to reduce the chance of missing defects due to low visibility. Examples in recent datasets include single-axis rotation [103], multiple illumination directions [104], and multiple view directions [45].

Synthetic datasets for inspection exist in the form of generic stochastic textures [105], outdoor maintenance scenes [30], multi-object pose estimation task [32], industrial part recognition [106], and surface defect recognition [45].

We present a dual dataset consisting of a real and synthetic part, following the multi-view inspection setup described in [45]. Our dataset focuses on recognition difficulties arising from complex reflectance of different surface finish patterns that may cause defect curtaining.

### 7.1. Real Dataset

**Figure 19:**
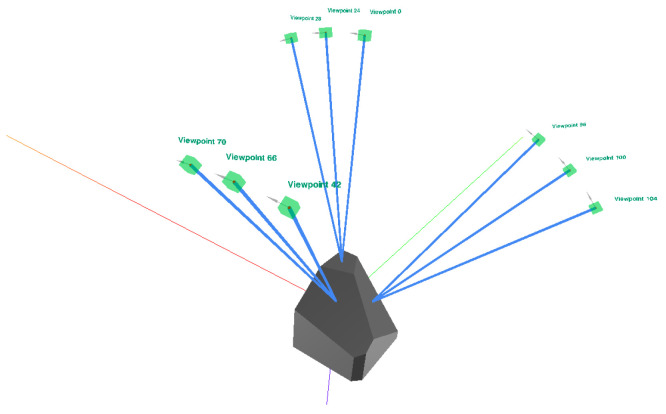
Visualization of the viewpoints used for inspection. The blue line shows the optical axis of the camera and has the length of the focusing distance.

The real dataset was acquired using a setup consisting of a robot manipulator, a matrix grayscale camera, a diffuse ring light, and an acquisition table. A thick black curtain removed any influence of the environment illumination or reflection from within the acquisition box. The manipulator was used to position the camera and illumination onto predefined viewpoints. The acquisition table was a flat surface covered in diffuse black velvet, and the acquired object was manually placed on top. The acquisition viewpoints were defined using V-POI [107] as an inspection plan, relative to the 3D model of the acquired object. Our acquisition plan consisted of viewpoints focused on the center of each inspected object face (faces A, B, and C), at angles 0°, 10° and 20° from the respective surface normal, as visualized in Figure 19. The viewpoints were at a distance of 140 mm from the object’s surface, in optical focus, and acquired in the size 2448×2050.

To minimize the placement error between the expected and actual position of the object, the operator must manually perform object positioning. This routine uses a predefined reference viewpoint—in our case, the viewpoint was perpendicular to the C-face—and a synthetic image of the object was taken from it. The physical camera was placed into the position defined by the reference viewpoint, and the reference synthetic image was overlaid. The object was then aligned manually to make the observed image match the reference image. For this procedure to be appropriate, either the synthetic image must contain image distortion introduced by the lens or the real image must be undistorted.

The real dataset consists of images of the test bodies defined in Section 3, acquired using the setup above and manually annotated using LabelMe [108]. The images were resized to a size of 1224×1025 so as to reduce the downstream costs while maintaining meaningful defect appearance clarity. The dataset contained 30 objects, 20 of which were defective, and with nine viewpoints per object. Overall this amounts to 270 annotated images, 180 of which display defects. The dataset was split into train-val-test sets. The test set contained 10 non-defective objects and 10 defective objects. The train and validation sets contained the remaining 10 defective objects, where only one viewpoint per object was used for the validation set.

### 7.2. Synthetic Dataset

The synthetic dataset generation environment described in Section 2 was developed to match the physical acquisition setup. The dataset generator is an automated procedure which uses object, texture, and defect specifications for generating annotated synthetic data in multiple stages. The first stage of the dataset generator is geometric defecting, as described in Section 6, which generates and applies defects to the original object geometry. We generated 30 defective synthetic object instances through stochastic sampling of the defect locations and specifications defined in Table 3. The defect locations were sampled independently over the inspected surfaces using uniform barycentric face sampling. Finally, we grouped our generated defects into the classes ’dent’ and ’scratch’.

The second stage produced texture images of the object surface using the methods introduced in Section 5. For each generated texture instance, the texture parameters were sampled at random from the values listed in Table 4, thus producing controlled surface appearance variations. The ranges were chosen empirically by observing differences in the physical samples to model plausible variations in the textures, such as milling at different translational speeds or amplitudes of parallel and orthogonal tool vibrations. For each of the three surface finishing methods, we generated 5 different texture instances.

The third stage combined the pre-generated synthetic objects and textures to produce annotated inspection images, as described in Section 2. A set of synthetic image-mask pairs were rendered for every synthetic object, following the same pre-defined inspection plan. Before rendering, each object surface was assigned one of the pre-generated textures at random. Additionally, each time a texture was assigned, it was randomly rotated by an angle in the range $\left[-15^{\circ },15^{\circ }\right]$ and translated by 5, 3, or 1 pixels for faces A, B, and C, respectively. To model different surface oxidation levels, for each synthetic object, we uniformly sampled the material’s roughness parameter from the range [0.05,0.3]. To produce a balanced dataset, we generated the same amount of image-mask pairs by re-using the correct object geometry instead of the defective ones.

Overall, the synthetic dataset consists of 30 correct and 30 defective object instances for each of the 10 physical objects. To remove the bias arising from the location and choice of defects when comparing the results between textures, we re-used the same 30 defective object geometries across all the physical objects. Each object was inspected using the same 9 viewpoints used in the pre-defined inspection plan, totaling in 5400 images, 2700 of which contained defects. The images were rendered using 256 SPP and 8 light bounces at a resolution of 1224×1025, with every object instance taking on average of 25 min to render with labels. The train-val-test set was produced, ensuring label balance in a ratio of 70:10:20 by splitting it along the dimension of object instances.

**Figure 20:**
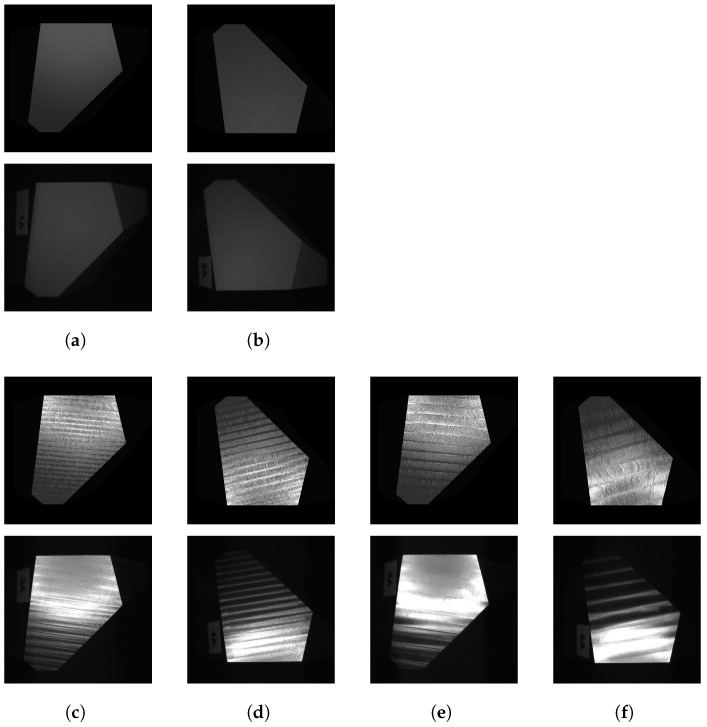

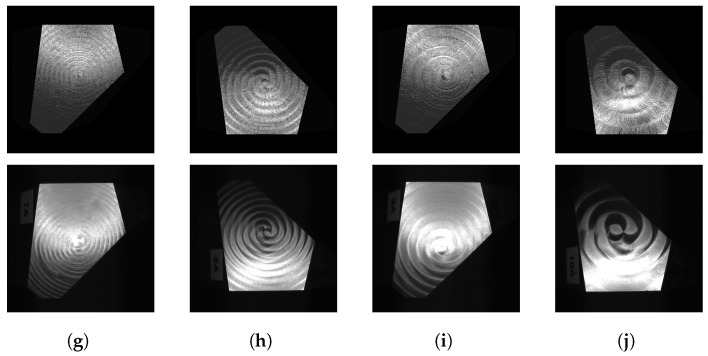
Non-defective representative image samples from the dual dataset. The upper image is synthetic, and the lower image is real. The images represent face A, viewed at a 20-degree angle from the perpendicular view. Selected synthetic images closely match the structures in real images. (**a**) Sandblasting, 2.5 bar. (**b**) Sandblasting, 6 bar. (**c**) Parallel milling: a head diameter of 4 mm and a radial depth of cut of 0.5. (**d**) Parallel milling: a head diameter of 4 mm and a radial depth of cut of 0.8. (**e**) Parallel milling: a head diameter of 8 mm and a radial depth of cut of 0.5. (**f**) Parallel milling: a head diameter of 8 mm and a radial depth of cut of 0.8. (**g**) Spiral milling: a head diameter of 4 mm and a radial depth of cut of 0.5. (**h**) Spiral milling: a head diameter of 4 mm and a radial depth of cut of 0.8. (**i**) Spiral milling: a head diameter of 8 mm and a radial depth of cut of 0.5. (**j**) Spiral milling: a head diameter of 8 mm and a radial depth of cut of 0.8.

A selection of images from our dataset is shown in Figure 20.

## 8. Pipeline Evaluation

To evaluate the quality of the pipeline, we have to evaluate the similarity of the data tht it produces to the real data acquired through inspection. Various metrics were proposed in recent works to estimate the quality of a dataset [109] through general dataset factors, such as annotation consistency, or class balance. However, these metrics usually provide limited insight into the data coverage of the dataset and ignore domain- and task-specific factors that could describe biases taken when collecting the dataset. Another approach for comparing the quality of synthetic datasets to the real ones is by measuring their utility for the given task through the model performance changes when using the synthetic data [15,44,45,106,110,111,112]. This, however, includes the inductive biases introduced by the model and training procedure selection, making it more difficult to estimate the quality of the dataset itself. We, thus, performed a more direct comparison of the datasets through image and data distribution similarities. When modeling a synthetic dataset, assumptions about the real world are made to increase the feasibility and economy of generating such datasets over the collection of more real data. To organize our evaluations, we proposed a distinction between a priori quality, evaluating the accuracy of the assumptions made before the data generation, and a posteriori quality, evaluating the accuracy of the generated synthetic data. To the best of the authors’ knowledge, this is a first evaluation of synthetic data quality that goes beyond the comparison of recognition model performances.

### 8.1. A Priori Quality

The a priori quality of a synthetic dataset quantifies the realism and precision of the environmental models used to generate the images compared to the target domain. It is reported through assumptions used to define parameters for the data generation and can be described prior to generating the final synthetic dataset. In our application, assumptions made to generate the dataset are tightly coupled to the physical processes used to manufacture and inspect the object. Using the same CAD model as used in manufacturing of the object, we guarantee the produced images will represent the correct geometry and restrict the data to the object of interest (Section 2.5). Using textures modeled to match the real surface measurements, we restricted the recognition task to the nominal patterns present in the real objects (Section 5). Where possible, the texture parameters were directly derived from the processing parameters. All parameters were sampled around the nominal values to increase model robustness towards variations in the surface texture (Section 7.2). Defect randomization contains ranges of parameters such as the expected defect dimensions and shapes that cover the defects that are present in the real objects, as well as the defects outside the observed range, for better generalization (Section 7.2). By using physics-based light simulation, we ensured that the images were produced realistically, holding the assumptions used for the material models and the rendering processes defined in Section 2.6. The simulated acquisition hardware (camera and light) uses the real acquisition hardware parameters to resemble the real inspection environment.

### 8.2. A Posteriori Quality

The a posteriori quality of a synthetic dataset describes the similarity of the generated data to the data collected in the target environment. This type of quality estimation relies on the established concepts of domain shifts actively used for methods of domain adaptation [113]. We categorized these qualities into domain similarity and task similarity.

#### 8.2.1. Domain Similarity

Domain similarity measures the similarity between the distributions of real and synthetic images to quantify the covariate shift. In our case, we measured the distance between the generated images and the collected real images acquired using different types of metrics.

Value-wise metrics compare the global distributions of intensities between images. Distributions of image intensities can be summarized using histograms and can be compared using a suitable metric [114].

Pattern-wise similarity compares the local patterns between the images. This type of comparison is affected by the scene structures, e.g., patterns of the surface finish, the light source shape, and the material reflectance function. Handcrafted image descriptors can be used to measure the similarities of specific aspects of patterns in images, such as pixel-wise closeness and alignment (e.g., the mean absolute error, root mean squared error, and cosine similarity) or statistical local similarity (e.g., SSIM [115], peak signal-to-noise ratio, Adapted Rand [116], and GLCM [117]). Learned feature extractors alleviate the need to manually select specific features in images and instead rely on a very diverse dataset and deep learning to train a general purpose feature space for pattern and shape comparisons (e.g., LPIPS [118] or FID [119]). However, due to inherent differences between natural (e.g., dogs, traffic, people, etc.) and technical images (e.g., surface inspection, manufacturing line, product appearance, etc.), the generality is still bound to the imaging context. To expand that context, one would need a large amount of representative technical images, which is not publicly available.

We evaluated the following metrics: Wasserstein distance between image histograms (HistWD), the mean absolute error (MAE), SSIM, and LPIPS, using ImageNet pretrained AlexNet [120]. We limited our study to a single representative metric from each aforementioned subgroup in regard to their popularity in image quality estimation, interpretability, and implementation availability. Our experiments will prove sufficient to discuss the influence and shortcomings of different metrics (Section 9.2). To make the results easier to read, the metrics were normalized to the range [0,1] and flipped when necessary to report the degree of similarity (see Table 5). Our reasoning is that metrics that report the highest differences in the dataset (excluding trivial metrics) account best for the small variations between the images in our datasets.

To compare our synthetic images to real ones, we needed to remove the influence of background noise and minor offsets that were present from the imprecision of the acquisition system and background scene structures. We first masked out all the regions that did not contain the observed object face, thus removing the influence of sharp edges between the dark background and the object. For each viewpoint, we constructed a common meta-mask from the intersection of face masks corresponding to that viewpoint. To reduce the difference in intensity levels, we manually estimated the parameters of a linear transformation to bring the histograms between matching real and synthetic images closer together. The minimal gray value in the real images was typically larger than 0, while the synthetic images also showed gray values down to 0. Thus, for each texture type, we add the mean minimal gray value observed in the real images to all synthetic images.

**Figure 21:**
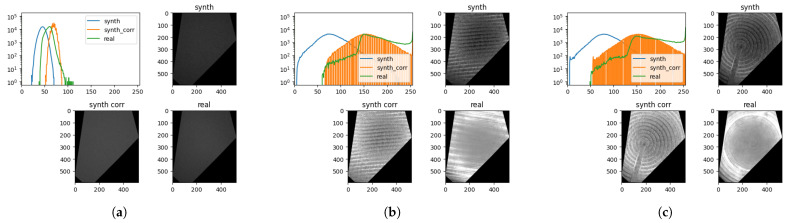
Examples of the differences made by intensity correction of synthetic images. Each example shows the image histograms, the original synthetic image (synth), the corrected synthetic image (synth_corr), and the closest matching real image (real). (**a**) Sandblasted, (**b**) parallel milling, and (**c**) spiral milling.

Pre-processing the data is an effective and efficient method to overcome some of the perceptual differences between domains. To jointly compensate for the illumination intensity and material absorption differences between synthetic and real data, we estimated an image multiplication factor. This method is justified, since the factor can be extracted from the integral of the rendering equation [49], assuming that the inter-reflectance of the surface mesostructures is negligible. The assumption holds for our case, since we use normal mapping for mesostructures, which does not produce inter-reflectance. The bias value was added to all the pixels to compensate for the ambient illumination caused by the low-intensity inter-reflections of the inspected object and the inspection environment. This can be observed in the image histograms as a gap of zeros between the illumination value 0 and the first next intensity value in the histogram. We estimated the bias by calculating the average gap size. The estimated factors and biases are as follows: (0.632,34) for sandblasted, (1.481,39) for parallel, and (1.526,33) for spiral milling. In Figure 21, examples of the intensity correction and the effect it has on the synthetic images are shown. Finally, since the synthetic pinhole camera model does not include blurring and imperfections of the real camera, we artificially blurred the synthetic images using defocus blur [121] with a radius of 1.0.

The synthetic data do not model an exact 1:1 replica of the texture found on a single real object, but it models stochastic variations of it. Therefore, we measured the distance to the closest synthetic sample, similarly to a one-sided Chamfer distance. When evaluating the metrics, for each viewpoint, we evaluated the metric between the real image and all the available synthetic object instances and selected the value representing the highest similarity (lowest distance). In Table 5, for each texture type, we reported the metric value averaged across all viewpoints and objects sharing the same texture type.

#### 8.2.2. Task Similarity

Task similarity is affected by concept shift and estimates how well the task function, mapping inputs to labels, obtained on synthetic data, transfers to the target dataset. In our case, we measured how well an image semantic segmentation model trained on synthetic data generalizes to the real data. In the field of domain adaptation [113], a common approach is to report the difference in task metrics. As discussed in [122], this approach measures the difference in biases present in the two datasets, which can be interpreted as domain shifts when working with datasets acquired from different environments.

We trained a UNet [123] with a ResNet-50 [124] backbone from five random parameter initializations per experiment and reported the top obtained result so as to reduce the bias of weight initialization. The model was trained to perform semantic segmentation through pixel classification into the classes background, dent, and scratch. Since the non-background classes are highly underrepresented, we observed the need to use class weighting, which we empirically found to be (1,1.5,1.5) for the real and (1,2,2) for the synthetic datasets. We optimized the models by using the AdamW optimizer [125] with a learning rate of $10^{-4}$ and a $L_{2}$ weight regularization of $10^{-5}$, for a maximum of 1000 epochs, with early stopping at validation loss convergence upon five consecutive validations without at least a $10^{-2}$ relative improvement. For model fine-tuning, we used the learning rate of $10^{-5}$ using the real train and validation splits. For memory efficiency, during training, we extracted random image crops of a size of 256×256, and during the evaluation, we ended up splitting the images into a 3×3 grid of patches of a size of 416×352. All images were zero padded to ensure dimension divisibility correctness when evaluating the UNet and image patching. To speed up model training, we centered the images linearly between [−1,1].

Domain alignment through image rendering is a memory and compute expensive operation, thus we offloaded as much of the domain alignment as possible to online post-processing. We commenced by pre-processing the synthetic images using the factor and bias that were estimated for domain similarity, with a masked application applied to only the main face of interest in the image. To simulate the non-zero background, we artificially added Gaussian noise of amplitudes in the interval [0,5] to the background around the object. To simulate the vertical blooming effect caused by photon leakage within the saturated regions of the CCD sensor array, we extracted the maximum value of each image column, masked the column using a threshold of 0.95, blurred it by using a box filter of size 64, multiplied it by 0.02, and added it to the original image with clipping. Furthermore, manually annotated real images contain imprecision in masks, as they often cover the area around the rim of the defect. We emulated this by dilating the synthetic masks by 1 pixel. The synthetic images contain masks in regions that have mostly uniform value, and should be considered invisible. We pre-filtered the masks to remove defects with insufficient visibility. We empirically estimated the visibility as the difference between the mean intensity of the defect and the mean intensity of the surface ring around the defect, calculated as the difference between the defect mask, which was dilated two times, and the original defect mask. Defects are considered visible if the difference in means is over 0.05.

Data augmentation further increases the diversity of the dataset and helps to regularize the recognition model so as to consider the important features. To simulate the offsets of the camera, we used a random rotation of amplitude 15°. Illumination strength and object material diffusion variation can be jointly controlled using random exposure from the interval [−0.5,1.5]. To simulate the blurred background structures and impurities in the lens or the light carrying medium, we added Gaussian noise of amplitude in the range of [0,5] to the entire image and then blurred it by using defocus blur [121] of kernel sizes in the interval of [1,3]. Gaussian noise of amplitude in [0,5] was added once more to simulate the static noise of the real images. Finally, images can be randomly flipped horizontally to increase the texture–structure pair diversity. All the augmentations were performed with a probability of 50%. Finally, we performed intensity-biased random cropping [45] using a threshold of 15 to ensure production of crops that contained more visible structures in the image.

The results of image segmentation were collected and are displayed in Table 6. When computing the mean score, the contribution of the background class to the mean value was ignored, as it is the majority class that would skew the results away from the defect classes. To evaluate the concept shift, we compare common semantic segmentation metrics of the model generalization when trained on the real subset to the synthetic subset.

## 9. Discussion

### 9.1. Pipeline Controllability and Simplicity

The pipeline is very versatile and allows for the generation of a wide variety of texture variations needed for domain randomization. We show that, even in the extremely low-data scenario of this study, we can model and generate a large variety of samples with great similarity to the real data while keeping the pipeline interpretable for further improvements in texture and lighting. The physically linked parametrization of the individual modules allows defining elements using physical measurements, thus reducing the initial expensive search for viable parameters. While rendering is the most computationally demanding component, it can be easily parallelized.

The large number of parameters make it difficult to study the influence of different parameter sampling schemes on recognition performance. However, this pipeline opens the opportunity to design a higher level of abstraction for dataset design through the modeling of lower dimensional constraints over larger sets of parameters. While this is out of the scope of this paper, we strongly encourage its research in the future.

### 9.2. Domain Similarity

Visually comparing the synthetic and real domains, we observed similar reflection patterns across all three texture types, working both for denser and these patterns. The parallel or spiral patterns corresponded to the ones in the real images, with the striped reflections falling off consistently. The reflection of the light source shape closely matched that in the real images; however, it was prone to slight differences due to texture directions or center offsets. Additionally, the variations in the texture consistently models that of the real data, especially the semi-randomness of the milling patterns, where opposite side rings appear at times as the dominant pattern. However, there are noticeable differences between the images, as well. The synthetic images are much sharper than the real ones due to the missing blur occurring from the imperfections of real camera and offsets of its position in space. In addition, the synthetic materials are more reflective or less rough than the ones in real images, observed by the difference in average intensity gradient along the vertical axis (most visible in sandblasted). However, in the views observed from the views perpendicular to the surface, the intensity drift is not visible. This might require a further study on the viability of existing material BRDF models and the need for finer-grain textures or modifications of the rendering algorithm.

Measuring texture similarity is a difficult problem due to surface and acquisition imperfections being present in the real data. Camera blurring, surface imperfections, and other effects move away from the nominal texture without degrading its performance, thus producing falsely bad results. In addition, it is difficult to generate a nominal image that perfectly aligns with the acquired image due to camera–object and texture–object alignments. In this work, we instead relied on methods that compare global or local pixel statistics of textures to remove alignment. From the results in Table 5, we observed that the sandblasted textures have the highest degree of similarity compared to the parallel and spiral milling textures. This result is related to the simplicity of modeling the sandblasted texture, as the texture appears as mostly uniform noise. More structured textures, such as those of milling, are more difficult to replicate, as they have significantly more parameters that interplay to produce unique patterns.

Comparing the values of metrics is difficult; even though they are bounded between [0,1] and might measure similar properties of images, they do not share the same linearity of response to changes in inputs. A separate study is required to compare which physical properties different metrics attempt measuring over additional datasets. Since such a study targets a different task, it requires different definitions of the dataset parameters, which is out of the scope of this study.

### 9.3. Task Similarity and Recognition Performance

The defect recognition results in Table 6 show very similar performances in all experiments, except when the model trained on the synthetic domain is evaluated directly on the real domain. The similarity of results, even after fine-tuning, indicates that the models are saturated in their capacity to solve the task. The low generalization to the real domain indicates that there exists a misalignment between the synthetic and real data distributions, both in the form of a covariate shift (as observed by similarity metrics) and concept shift. The results obtained from the models trained on all textures do not surpass the performance on the highest performing sandblasted texture, leading us to the conclusion that training a model on multiple textures at once does not give any benefit over training a separate model for each texture.

When inspecting the model predictions over the datasets, we noticed some patterns. All models tend to predict masks that are slightly wider than the label. Most false positives come from the milled textures, in the lines where the two milling path circles overlap and along the exit lines. In sandblasted textures, tiny glints from the surface are often falsely predicted as defected. False positives are also present in minor deviations of an object’s shape, such as edge scuffing, beveling, deeper sandblasting holes, etched signatures, on paper label stickers, and where the milled pattern has a high contrast. Models also tend to predict defects on out-of-focus faces, which the annotator left as unannotated due to heavy blurring or unrecognizability. When predicting scratches, the models prefer areas where the texture has lower contrast and defect stand out. Some scratches that are not close to being horizontal or vertical are not predicted, even though the visibility is similar to the predicted ones, meaning the recognition models need more filters in lower levels or more explicit rotation invariance. Overall, it seems a higher resolution might help the model distinguish between defect and texture patterns.

### 9.4. Influence of Domain Similarity on Task Performance

The largest drop in recognition performance is on milled textures, which is consistent with their lower domain similarity when compared to the sandblasted textures. Furthermore, SSIM and LPIPS seem to be linearly correlated to the best obtained recognition performance. If this were true, we could use these metrics when iteratively improving the synthetic data to reduce the domain gap at its source, the data simulation, or the generation process. This forms the questions of whether these metrics are indeed predictive of the domain gap that impedes the recognition performance, which other metrics have a similar property, and why MAE and histogram comparisons do not seem to be. We find these questions are out of the scope of this paper, and we leave their careful treatment for a future study.

## 10. Conclusions

The presented method is capable of generating synthetic data for structured surface textures for a metal object by using a single sample of a real surface and knowledge about the manufacturing process. It allows precise control over its parameters, and the parameters are modeled to represent physically measurable or intuitively approximate features. The generated data offer the highest degree of realism, yet, for the studied textures, in terms of patterns, the intensity distributions and defects vary. Furthermore, the presented method is capable of generating synthetic data at scale, with support from the physically plausible variations in texture and defect parameters. The separation of modeling steps gave rise to the concept of scales decomposition, and provides a clear backbone on how further developments can improve and extend the capabilities of the pipeline. The decomposition of the dataset quality measurements allowed us to compare its similarity between the synthetic and real datasets as a whole while separating the similarity of the domain from the similarity of the task. The uncovered predictive trend that similarity metrics have on defect detection performance offers an efficient measurement technique for iterative synthetic data development. Finally, the published dual dataset serves the community by offering missing data for inspection of structured surfaces, facilitates domain adaptation research, and contributes a benchmark for the development of industrial inspection models.

## Figures and Tables

**Table 1:** Parameter settings for surface processing.

Technique	Parameter	Values
milling	Milling head’s diameter	4 mm, 8 mm
	Radial depth of the cut	0.2, 0.5, 0.8
	Path	parallel, spiral
sandblasting	Pressure	2.5 bar, 6 bar

**Table 2:** Overview of all parameters needed for the milling model, including their meaning and choices thereof. Choose conf=3 for normally distributed parameters. Known parameters are highlighted (Table 1).

	Notation	Definition	Meaning	Distribution	Parameter	Values
Pattern	$c_{k}$	rings’ center points	determined by tool path	$N\left({\tilde{c}}_{k},\Sigma_{c}\right)$	$\Sigma_{c}\in R_{>0}^{2\times 2}$	${\left(\Sigma_{c}\right)}_{ii}=0.01\;\cdot \;\delta /\mathrm{conf}$, i=1,2
	*d*	diameter of ring	**diameter of milling head**		$d\in R_{>0}$	d∈{4,8}mm
	α	defines ρ (distance between neighboring tool paths)	**amount of overlap of neighboring tool paths**		α∈(0,1)	α∈{0.2,0.5,0.8}
	γ	increase distance between neighboring tool paths	distance between blade and outer edge of tool		γ∈(0,α)	γ=0.04
	δ	distance between center points	depends on feed rate and tool’s rotational speed		$\delta \in R_{>0}$	δ=0.09 mm
	ϵ	amount of rings with changed order			ϵ∈[0,1]	ϵ=0.01
Appearance	$w_{k}^{-}$	width of indentation	width of cutting edge	$N\left(\mu_{w^{-}},\sigma_{w^{-}}\right)$	$\mu_{w^{-}}\in \left(0,d\;/\;2\right)$ $\sigma_{w^{-}}\in R_{>0}$	$\mu_{w^{-}}=0.05\;\mathrm{mm}$ $\sigma_{w^{-}}=0.025/\mathrm{conf}$
	$w_{k}^{+i}$	width of inner accumulation	depends on edges’ sharpness	$N\left(\mu_{w^{+i}},\sigma_{w^{+i}}\right)$	$\mu_{w^{+i}}\in \left[0,d\;/\;2-\mu_{w^{-}}\right)$ $\sigma_{w^{+i}}\in R_{>0}$	$\mu_{w^{+i}}=0.025\;\mathrm{mm}$ $\sigma_{w^{+i}}=0.01/\mathrm{conf}$
	$w_{k}^{+o}$	width of outer accumulation	depends on edges’ sharpness	$N\left(\mu_{w^{+o}},\sigma_{w^{+o}}\right)$	$\mu_{w^{+o}}\in R_{\geq 0}$ $\sigma_{w^{+o}}\in R_{>0}$	$\mu_{w^{+o}}=0.025\;\mathrm{mm}$ $\sigma_{w^{+o}}=0.01/\mathrm{conf}$
	$\phi_{k}$	tilting direction	depends on tool path		$\phi_{k}\in \left(-\pi ,\pi \right]$	computed by *c_k_*
	$l_{k}^{-}$, $h_{k}^{-}$	minimal/maximal scaling of indentation depth	cutting depth with tilting	$N\left(\mu_{{•}^{-}},\sigma_{{•}^{-}}\right)$	$\mu_{{•}^{-}}\in R_{\geq 0}$ $\sigma_{{•}^{-}}\in R_{\geq 0}$	$\mu_{l^{-}}=0.7$, $\mu_{h^{-}}=1$ $\sigma_{{•}^{-}}=0.8/\mathrm{conf}$
	$l_{k}^{+i}$, $h_{k}^{+i}$	minimal/maximal scaling of inner accumulation height	depends on edges’ sharpness	$N\left(\mu_{{•}^{+i}},\sigma_{{•}^{+i}}\right)$	$\mu_{{•}^{+i}}\in R_{\geq 0}$ $\sigma_{{•}^{+i}}\in R_{\geq 0}$	$\mu_{l^{+i}}=0$, $\mu_{h^{+i}}=0.2$ $\sigma_{{•}^{+i}}=0.5/\mathrm{conf}$
	$l_{k}^{+o}$, $h_{k}^{+o}$	minimal/maximal scaling of outer accumulation height	depends on edges’ sharpness	$N\left(\mu_{{•}^{+o}},\sigma_{{•}^{+o}}\right)$	$\mu_{{•}^{+o}}\in R_{\geq 0}$ $\sigma_{{•}^{+o}}\in R_{\geq 0}$	$\mu_{l^{+o}}=0.2$, $\mu_{h^{+o}}=0.5$ $\sigma_{{•}^{+o}}=0.5/\mathrm{conf}$
	$\lambda_{k}$	number of sine curves		P(λ)	λ∈N	λ=50
	$\tau_{k_{j}}$	frequency of sine curves		P(τ)	τ∈N	τ=50
	$\xi_{k_{j}}$	shift of sine curves		$U\left(-\pi ,\pi \right)$		
Interaction	$a_{k}$	minimal value convex combination		$U\left(a^{\min},a^{\max}\right)$	$a^{\min}\in \left[0,1\right]$ $a^{\max}\in \left[a^{\min},1\right]$	$a^{\min}=0$ $a^{\max}=0.3$
	$b_{k}$	maximal value convex combination		$U\left(b^{\min},b^{\max}\right)$	$b^{\min}\in \left[0,1\right]$ $b^{\max}\in \left[b^{\min},1\right]$	$b^{\min}=0.1$ $b^{\max}=0.4$

**Table 3:** Defect quantities and specification ranges obtained from approximate measurements of their correspondents in physical samples with a gentle increase in ranges to model the expected unobserved defects.

Parameter	Small Dent	Big Dent	Flat Scratch	Curvy Scratch
Quantity	5	3	2	2
Diameter	[0.02, 0.2]	[0.2, 1.0]	[0.02, 0.2]	[0.02, 0.1]
Elongation	[1, 2]	[1, 4]	-	-
Depth	[0.05, 0.2]	[0.2, 1.0]	-	-
Path length	-	-	[5, 20]	[10, 20]
Step size	-	-	0.1	1.0

**Table 4:** Texture parameter ranges used to modify the default values (highlighted) given in Table 2. The standard deviations were chosen by the 3σ rule such that the desired ranges of the random variables are obtained. Hence, we set conf=3.

Parameter	Set of Values
Ring center points noise ($\Sigma_{c}$)	$\left\{0.01,0.05,0.1,0.2,0.4,0.6,0.8\right\}\;\cdot \;\delta \;/\;\mathrm{conf}$
Ring distance (δ)	$\left\{0.7,0.75,0.8,0.85,0.9,0.95,1.0,1.05,1.1\right\}$
Ring flip probability (ϵ)	$\left\{0.01,0.05,0.1,0.2,0.3,0.4,0.5\right\}$
Ring width noise ($\sigma_{\omega^{-}}$)	$\left\{0.015,0.02,0.025,0.03,0.035\right\}\;\cdot \;1\;/\;\mathrm{conf}$
Ring depth noise ($\sigma_{l^{-}},\sigma_{h^{-}}$)	$\left\{0.4,0.6,0.8,1.0,1.2\right\}\;\cdot \;1\;/\;\mathrm{conf}$
Cosine curves number (λ)	$\left\{30,50,70\right\}$

**Table 5:** Domain similarities averaged within and across texture types, bound to interval [0,1]. Histogram WD, MAE, and LPIPS were inverted to be interpreted as the degree of similarity.

Method	Sandblasted	Parallel	Spiral	All
1—HistWD	0.980	0.932	0.942	0.946
1—MAE	0.807	0.681	0.655	0.696
SSIM	0.896	0.561	0.585	0.638
1—LPIPS	0.916	0.660	0.686	0.722

**Table 6:** Task similarities between texture groups. The domains represent the experiment training → testing domains, with the real (Re) and synthetic (Sy) domains. Fine-tuning (ft) was performed using real data after training on synthetic data.

Texture	Domains	mP	mR	mF1	mIoU
Sandblasted	Sy → Sy	57.0	53.1	54.7	37.7
	Sy → Re	34.0	64.3	41.7	26.3
	Sy + ft → Re	60.1	71.9	65.5	49.6
	Re → Re	57.9	61.9	59.1	42.6
Parallel	Sy → Sy	57.1	39.6	45.6	29.6
	Sy → Re	26.5	23.0	23.5	13.8
	Sy + ft → Re	52.6	33.4	38.3	23.9
	Re → Re	50.3	34.5	40.8	25.8
Spiral	Sy → Sy	59.5	39.7	47.6	31.3
	Sy → Re	35.2	22.1	26.4	15.5
	Sy + ft → Re	49.5	40.1	43.9	28.4
	Re → Re	50.6	43.1	46.2	30.7
All	Sy → Sy	59.4	46.0	51.5	34.7
	Sy → Re	32.2	31.3	31.6	18.9
	Sy + ft → Re	59.0	49.1	53.1	36.1
	Re → Re	59.0	48.2	52.6	35.8

## Data Availability

The SYNOSIS dataset used in this study is published and publicly available at: http://dx.doi.org/10.24406/fordatis/370.

## Abbreviations

The following abbreviations are used in this manuscript:

ADSN	Asymptotic Discrete Spot Noise
BRDF	Bidirectional Reflectance Distribution Function
CAD	Computer-Assisted Design
CCD	Charge-Coupled Device
GAN	Generative Adversarial Networks
LPIPS	Learned Perceptual Image Patch Similarity metric
MAE	Mean Absolute Error
ML	Machine Learning
SPP	Samples-per-pixel
SSIM	Structural Similarity metric

## References

[1] Gospodnetić P., Mosbach D., Rauhut M., Hagen H. Flexible Surface Inspection Planning Pipeline. Proceedings of the 6th International Conference on Control, Automation and Robotics (ICCAR).

[2] Wagenstetter M., Gospodnetic P., Bosnar L., Fulir J., Kreul D., Rushmeier H., Aicher T., Hellmich A., Ihlenfeldt S. Synthetically generated images for industrial anomaly detection. Proceedings of the IEEE International Conference on Emerging Technologies and Factory Automation.

[3] Nikolenko S.I. (2021). Synthetic Data for Deep Learning.

[4] Haselmann M., Gruber D.P. (2019). Pixel-Wise Defect Detection by CNNs without Manually Labeled Training Data. Appl. Artif. Intell..

[5] Schladitz K., Redenbach C., Barisin T., Jung C., Jeziorski N., Bosnar L., Fulir J., Gospodnetic P. Simulation of microstructures and machine learning. Proceedings of the CMDS14.

[6] Jain S., Seth G., Paruthi A., Soni U., Kumar G. (2020). Synthetic data augmentation for surface defect detection and classification using deep learning. J. Intell. Manuf..

[7] Zhang G., Cui K., Hung T.Y., Lu S. Defect-GAN: High-Fidelity Defect Synthesis for Automated Defect Inspection. Proceedings of the IEEE WACV.

[8] Wang R., Hoppe S., Monari E., Huber M. (2022). Defect Transfer GAN: Diverse Defect Synthesis for Data Augmentation. Proceedings of the 33rd British Machine Vision Conference 2022, BMVC 2022.

[9] Wei J., Shen F., Lv C., Zhang Z., Zhang F., Yang H. Diversified and Multi-Class Controllable Industrial Defect Synthesis for Data Augmentation and Transfer. Proceedings of the 2023 IEEE/CVF CVPRW.

[10] Schmedemann O., Schlodinski S., Holst D., Schüppstuhl T. Adapting synthetic training data in deep learning-based visual surface inspection to improve transferability of simulations to real-world environments. Proceedings of the Automated Visual Inspection and Machine Vision V. SPIE.

[11] Rogulsky S., Popovic N., Färber M. (2024). The Effects of Hallucinations in Synthetic Training Data for Relation Extraction. arXiv.

[12] Sun Y., Sheng D., Zhou Z., Wu Y. (2024). AI hallucination: Towards a comprehensive classification of distorted information in artificial intelligence-generated content. Humanit. Soc. Sci. Commun..

[13] Lee F., Hajisharif S., Johnson E. (2025). The ontological politics of synthetic data: Normalities, outliers, and intersectional hallucinations. Big Data Soc..

[14] Dahmen T., Trampert P., Boughorbel F., Sprenger J., Klusch M., Fischer K., Kübel C., Slusallek P. (2019). Digital reality: A model-based approach to supervised learning from synthetic data. AI Perspect..

[15] Tsirikoglou A., Eilertsen G., Unger J. (2020). A Survey of Image Synthesis Methods for Visual Machine Learning. Comput. Graph. Forum..

[16] Varol G., Romero J., Martin X., Mahmood N., Black M.J., Laptev I., Schmid C. Learning from synthetic humans. Proceedings of the IEEE Conference on Computer Vision and Pattern Recognition.

[17] Park D., Ramanan D. Articulated pose estimation with tiny synthetic videos. Proceedings of the IEEE Conference on Computer Vision and Pattern Recognition Workshops.

[18] Fabbri M., Lanzi F., Calderara S., Palazzi A., Vezzani R., Cucchiara R. Learning to detect and track visible and occluded body joints in a virtual world. Proceedings of the European conference on computer vision (ECCV).

[19] Kortylewski A., Schneider A., Gerig T., Egger B., Morel-Forster A., Vetter T. (2018). Training deep face recognition systems with synthetic data. arXiv.

[20] Dosovitskiy A., Ros G., Codevilla F., Lopez A., Koltun V. CARLA: An open urban driving simulator. Proceedings of the Conference on Robot Learning, PMLR.

[21] Shafaei A., Little J.J., Schmidt M. (2016). Play and learn: Using video games to train computer vision models. arXiv.

[22] Hurl B., Czarnecki K., Waslander S. (2019). Precise synthetic image and lidar (presil) dataset for autonomous vehicle perception. Proceedings of the 2019 IEEE Intelligent Vehicles Symposium (IV).

[23] Tremblay J., To T., Sundaralingam B., Xiang Y., Fox D., Birchfield S. (2018). Deep object pose estimation for semantic robotic grasping of household objects. arXiv.

[24] NVIDIA Isaac—The Accelerated Platform for Robotics and AI. https://www.nvidia.com/en-us/deep-learning-ai/industries/robotics/.

[25] Khan S., Phan B., Salay R., Czarnecki K. ProcSy: Procedural Synthetic Dataset Generation Towards Influence Factor Studies Of Semantic Segmentation Networks. Proceedings of the CVPR Workshops.

[26] Buls E., Kadikis R., Cacurs R., Ārents J. (2019). Generation of synthetic training data for object detection in piles. Proceedings of the Eleventh International Conference on Machine Vision (ICMV 2018).

[27] Greff K., Belletti F., Beyer L., Doersch C., Du Y., Duckworth D., Fleet D.J., Gnanapragasam D., Golemo F., Herrmann C. Kubric: A scalable dataset generator. Proceedings of the IEEE/CVF Conference on Computer Vision and Pattern Recognition.

[28] Moonen S., Vanherle B., de Hoog J., Bourgana T., Bey-Temsamani A., Michiels N. CAD2Render: A Modular Toolkit for GPU-accelerated Photorealistic Synthetic Data Generation for the Manufacturing Industry. Proceedings of the IEEE/CVF Winter Conference on Applications of Computer Vision.

[29] Wieler M., Hahn T. Weakly supervised learning for industrial optical inspection. Proceedings of the DAGM Symposium.

[30] Bao T., Chen J., Li W., Wang X., Fei J., Wu L., Zhao R., Zheng Y. MIAD: A maintenance inspection dataset for unsupervised anomaly detection. Proceedings of the IEEE/CVF International Conference on Computer Vision.

[31] Boikov A., Payor V., Savelev R., Kolesnikov A. (2021). Synthetic data generation for steel defect detection and classification using deep learning. Symmetry.

[32] De Roovere P., Moonen S., Michiels N., Wyffels F. (2022). Dataset of Industrial Metal Objects. arXiv.

[33] de Hoog J., Grimard G., Bourgana T., Michiels N., Moonen S., De Geest R., Bey-Temsamani A. CAD2X—A Complete, End-to-End Solution for Training Deep Learning Networks for Industrial Applications. Proceedings of the 2023 Smart Systems Integration Conference and Exhibition (SSI).

[34] Saiz F.A., Alfaro G., Barandiaran I., Garcia S., Carretero M., Graña M. Synthetic Data Set Generation for the Evaluation of Image Acquisition Strategies Applied to Deep Learning Based Industrial Component Inspection Systems. Proceedings of the CEIG.

[35] Yang B., Liu Z., Duan G., Tan J. (2021). Mask2Defect: A prior knowledge-based data augmentation method for metal surface defect inspection. IEEE Trans. Ind. Inform..

[36] Abubakr A.G., Jovancevic I., Mokhtari N.I., Abdallah H.B., Orteu J.J. On learning deep domain-invariant features from 2D synthetic images for industrial visual inspection. Proceedings of the Fifteenth International Conference on Quality Control by Artificial Vision, SPIE.

[37] Kim A., Lee K., Lee S., Song J., Kwon S., Chung S. (2022). Synthetic Data and Computer-Vision-Based Automated Quality Inspection System for Reused Scaffolding. Appl. Sci..

[38] Sauer S., Borkar M., Sasidharan D., Dunker T. Model-based visual inspection with machine learning methods using simulation of the expected camera view. Proceedings of the Workshop on “Generating Synthetic Image Data for AI” at the KI 2022.

[39] Raymond B., Guennebaud G., Barla P. (2016). Multi-scale rendering of scratched materials using a structured SV-BRDF model. ACM Trans. Graph. (TOG).

[40] Bosnar L., Saric D., Dutta S., Weibel T., Rauhut M., Hagen H., Gospodnetic P. Image Synthesis Pipeline for Surface Inspection. Proceedings of the LEVIA’20: Leipzig Symposium on Visualization in Applications 2022.

[41] Gospodnetic P. (2021). Visual Surface Inspection Planning for Industrial Applications.

[42] Bosnar L., Rauhut M., Hagen H., Gospodnetic P. Texture Synthesis for Surface Inspection. Proceedings of the LEVIA’20: Leipzig Symposium on Visualization in Applications 2022.

[43] Bosnar L., Hagen H., Gospodnetic P. (2023). Procedural Defect Modeling for Virtual Surface Inspection Environments. IEEE Comput. Graph. Appl..

[44] Schmedemann O., Baaß M., Schoepflin D., Schüppstuhl T. (2022). Procedural synthetic training data generation for AI-based defect detection in industrial surface inspection. Procedia CIRP.

[45] Fulir J., Bosnar L., Hagen H., Gospodnetić P. Synthetic Data for Defect Segmentation on Complex Metal Surfaces. Proceedings of the 2023 IEEE/CVF Conference on Computer Vision and Pattern Recognition Workshops (CVPRW).

[46] Dorsey J., Rushmeier H., Sillion F. (2010). Digital Modeling of Material Appearance.

[47] Shannon C. (1949). Communication in the Presence of Noise. Proc. IRE.

[48] Greenberg D.P., Torrance K.E., Shirley P., Arvo J., Lafortune E., Ferwerda J.A., Walter B., Trumbore B., Pattanaik S., Foo S.C. A framework for realistic image synthesis. Proceedings of the 24th Annual Conference on Computer Graphics and Interactive Techniques.

[49] Kajiya J.T. The rendering equation. Proceedings of the 13th Annual Conference on Computer Graphics and Interactive Techniques.

[50] Pharr M., Jakob W., Humphreys G. (2016). Physically Based Rendering: From Theory to Implementation.

[51] Beaune F., Tovagliari E., Barrancos L., Agyemang S., Basu S., Bhutani M., Bosnar L., Brune R., Chan M., Costa J.M.M. (2019). Appleseed, Version 2.1.0-Beta.

[52] Walter B., Marschner S.R., Li H., Torrance K.E. (2007). Microfacet Models for Refraction through Rough Surfaces. Render. Tech..

[53] Kulla C., Conty A. Revisiting physically based shading at Imageworks. Proceedings of the Siggraph Course, Physically Based Shading.

[54] Turquin E. (2019). Practical Multiple Scattering Compensation for Microfacet Models. https://blog.selfshadow.com/publications/turquin/ms_comp_final.pdf.

[55] Mikkelsen M.S. (2010). Bump mapping unparametrized surfaces on the gpu. J. Graph. GPU Game Tools.

[56] Guerrero P., Hašan M., Sunkavalli K., Měch R., Boubekeur T., Mitra N.J. (2022). Matformer: A generative model for procedural materials. arXiv.

[57] Adobe Substance 3D Assets. https://substance3d.adobe.com/assets.

[58] Ebert D.S., Musgrave F.K., Peachey D., Perlin K., Worley S. (2003). Texturing & Modeling: A Procedural Approach.

[59] Dong Z., Walter B., Marschner S., Greenberg D.P. (2015). Predicting appearance from measured microgeometry of metal surfaces. ACM Trans. Graph..

[60] Bosnar L., Hagen H., Gospodnetic P. (2021). Texture Synthesis for Automated Visual Surface Inspection Planning.

[61] Hormann K., Lévy B., Sheffer A. Mesh parameterization: Theory and practice. Proceedings of the ACM SIGGRAPH 2007 Courses.

[62] (1998). Geometrical Product Specification (GPS)—Surface Imperfections—Terms, Definitions and Parameters.

[63] Jeziorski N., Redenbach C. (2024). Stochastic Geometry Models for Texture Synthesis of Machined Metallic Surfaces: Sandblasting and Milling. J. Math. Ind..

[64] Vivo P.G., Lowe J. (2015). The Book of Shaders. Dosegljivo. https://thebookofshaders.com.

[65] Dong J., Liu J., Yao K., Chantler M., Qi L., Yu H., Jian M. (2020). Survey of procedural methods for two-dimensional texture generation. Sensors.

[66] Deguy S. (2019). The New Age of Procedural Texturing.

[67] Dostal C., Yamafune K. (2018). Photogrammetric texture mapping: A method for increasing the Fidelity of 3D models of cultural heritage materials. J. Archaeol. Sci. Rep..

[68] Ritz M., Breitfelder S., Santos P., Kuijper A., Fellner D.W. (2019). Seamless and non-repetitive 4D texture variation synthesis and real-time rendering for measured optical material behavior. Comput. Vis. Media.

[69] Jetchev N., Bergmann U., Vollgraf R. (2016). Texture synthesis with spatial generative adversarial networks. arXiv.

[70] Bergmann U., Jetchev N., Vollgraf R. (2017). Learning texture manifolds with the periodic spatial GAN. arXiv.

[71] Zeltner T., Rousselle F., Weidlich A., Clarberg P., Novák J., Bitterli B., Evans A., Davidovič T., Kallweit S., Lefohn A. (2023). Real-Time Neural Appearance Models. arXiv.

[72] Raad L., Davy A., Desolneux A., Morel J.M. (2017). A survey of exemplar-based texture synthesis. arXiv.

[73] Galerne B., Gousseau Y., Morel J.M. (2011). Random Phase Textures: Theory and Synthesis. IEEE Trans. Image Process..

[74] Heeger D., Bergen J. Pyramid-based texture analysis/synthesis. Proceedings of the International Conference on Image Processing.

[75] Briand T., Vacher J., Galerne B., Rabin J. (2014). The Heeger & Bergen Pyramid Based Texture Synthesis Algorithm. Image Process. Line.

[76] Portilla J., Simoncelli E. (2000). A Parametric Texture Model Based on Joint Statistics of Complex Wavelet Coefficients. Int. J. Comput. Vis..

[77] Vacher J., Briand T. (2021). The Portilla-Simoncelli Texture Model: Towards Understanding the Early Visual Cortex. Image Process. Line.

[78] Efros A., Freeman W. (2001). Image Quilting for Texture Synthesis and Transfer. Comput. Graph..

[79] Raad L., Galerne B. (2017). Efros and Freeman Image Quilting Algorithm for Texture Synthesis. Image Process. Line.

[80] Efros A.A., Leung T.K. Texture synthesis by non-parametric sampling. Proceedings of the Seventh IEEE International Conference on Computer Vision.

[81] Guehl P., Allègre R., Dischler J.M., Benes B., Galin E. (2020). Semi-Procedural Textures Using Point Process Texture Basis Functions. Comput. Graph. Forum.

[82] Hu Y., He C., Deschaintre V., Dorsey J., Rushmeier H. (2022). An inverse procedural modeling pipeline for svbrdf maps. ACM Trans. Graph..

[83] Zhu J., Zhao S., Xu Y., Meng X., Wang L., Yan L.Q. (2022). Recent advances in glinty appearance rendering. Comput. Vis. Media.

[84] Chermain X., Claux F., Mérillou S. (2019). Glint Rendering based on a Multiple-Scattering Patch BRDF. Comput. Graph. Forum.

[85] Groover M.P. (2020). Fundamentals of Modern Manufacturing: Materials, Processes, and Systems.

[86] Childs T.H., Maekawa K., Obikawa T., Yamane Y. (2000). Metal Machining: Theory and Applications.

[87] Felhő C., Karpuschewski B., Kundrák J. (2015). Surface Roughness Modelling in Face Milling. Procedia CIRP.

[88] Felhő C., Kundrák J. (2018). Effects of Setting Errors (Insert Run-Outs) on Surface Roughness in Face Milling When Using Circular Inserts. Machines.

[89] Kundrák J., Felhő C., Nagy A. (2022). Analysis and Prediction of Roughness of Face Milled Surfaces using CAD Model. Manuf. Technol..

[90] Hadad M., Ramezani M. (2016). Modeling and analysis of a novel approach in machining and structuring of flat surfaces using face milling process. Int. J. Mach. Tools Manuf..

[91] Oranli E., Gungoren N., Astaraee A.H., Maleki E., Bagherifard S., Guagliano M. (2023). Numerical and experimental analysis of sand blasting on polymeric substrates. Forces Mech..

[92] Tang B., Chen L., Sun W., Lin Z.k. (2023). Review of surface defect detection of steel products based on machine vision. IET Image Process..

[93] Song K., Yan Y. (2013). A noise robust method based on completed local binary patterns for hot-rolled steel strip surface defects. Appl. Surf. Sci..

[94] Fu G., Sun P., Zhu W., Yang J., Cao Y., Yang M.Y., Cao Y. (2019). A deep-learning-based approach for fast and robust steel surface defects classification. Opt. Lasers Eng..

[95] Severstal: Steel Defect Detection–Kaggle. https://www.kaggle.com/c/severstal-steel-defect-detection.

[96] Lv X., Duan F., Jiang J.j., Fu X., Gan L. (2020). Deep Metallic Surface Defect Detection: The New Benchmark and Detection Network. Sensors.

[97] Tabernik D., Šela S., Skvarč J., Skočaj D. (2020). Segmentation-based deep-learning approach for surface-defect detection. J. Intell. Manuf..

[98] Božič J., Tabernik D., Skočaj D. (2021). KolektorSDD2: Mixed supervision for surface-defect detection: From weakly to fully supervised learning. Comput. Ind..

[99] Zhang Z., Yu S., Yang S., Zhou Y., Zhao B. (2021). Rail-5k: A Real-World Dataset for Rail Surface Defects Detection. arXiv.

[100] Tianchi (2018). Aluminum Profile Surface Flaw Recognition Dataset. Guangdong Industrial Manufacturing Big Data Innovation Competition. https://tianchi.aliyun.com/dataset/140666.

[101] Bergmann P., Fauser M., Sattlegger D., Steger C. MVTec AD—A Comprehensive Real-World Dataset for Unsupervised Anomaly Detection. Proceedings of the 2019 IEEE/CVF Conference on Computer Vision and Pattern Recognition (CVPR).

[102] Mishra P., Verk R., Fornasier D., Piciarelli C., Foresti G.L. VT-ADL: A Vision Transformer Network for Image Anomaly Detection and Localization. Proceedings of the 2021 IEEE 30th International Symposium on Industrial Electronics (ISIE).

[103] Schlagenhauf T., Landwehr M. (2021). Industrial machine tool component surface defect dataset. Data Brief..

[104] Honzátko D., Türetken E., Bigdeli S.A., Dunbar L.A., Fua P. (2021). Defect segmentation for multi-illumination quality control systems. Mach. Vis. Appl..

[105] Matthias W., Tobias H. DAGM: Weakly Supervised Learning for Industrial Optical Inspection. Proceedings of the DAGM Symposium.

[106] Zhu X., Bilal T., Mårtensson P., Hanson L., Björkman M., Maki A. Towards Sim-to-Real Industrial Parts Classification with Synthetic Dataset. Proceedings of the 2023 IEEE/CVF Conference on Computer Vision and Pattern Recognition Workshops (CVPRW).

[107] Gospodnetic P., Rauhut M., Hagen H. Surface Inspection Planning Using 3D Visualization. Proceedings of the LEVIA 2020: Leipzig Symposium on Visualization In Applications 2020.

[108] Wada K. (2021). Labelme: Image Polygonal Annotation with Python.

[109] Gong Y., Liu G., Xue Y., Li R., Meng L. (2023). A survey on dataset quality in machine learning. Inf. Softw. Technol..

[110] Wood E., Baltrušaitis T., Hewitt C., Dziadzio S., Cashman T.J., Shotton J. Fake It Till You Make It: Face Analysis in the Wild Using Synthetic Data Alone. Proceedings of the IEEE/CVF International Conference on Computer Vision (ICCV).

[111] Tremblay J., Prakash A., Acuna D., Brophy M., Jampani V., Anil C., To T., Cameracci E., Boochoon S., Birchfield S. Training Deep Networks with Synthetic Data: Bridging the Reality Gap by Domain Randomization. Proceedings of the 2018 IEEE/CVF Conference on Computer Vision and Pattern Recognition Workshops (CVPRW).

[112] Gaidon A., Wang Q., Cabon Y., Vig E. Virtual Worlds as Proxy for Multi-object Tracking Analysis. Proceedings of the 2016 IEEE Conference on Computer Vision and Pattern Recognition (CVPR).

[113] Farahani A., Voghoei S., Rasheed K., Arabnia H.R. A Brief Review of Domain Adaptation. Proceedings of the Transactions on Computational Science and Computational Intelligence.

[114] Ma Y., Gu X., Wang Y. (2010). Histogram similarity measure using variable bin size distance. Comput. Vis. Image Underst..

[115] Wang Z., Bovik A., Sheikh H., Simoncelli E. (2004). Image quality assessment: From error visibility to structural similarity. IEEE Trans. Image Process..

[116] Arganda-Carreras I., Turaga S.C., Berger D.R., Cireşan D., Giusti A., Gambardella L.M., Schmidhuber J., Laptev D., Dwivedi S., Buhmann J.M. (2015). Crowdsourcing the creation of image segmentation algorithms for connectomics. Front. Neuroanat..

[117] Haralick R.M., Shanmugam K., Dinstein I. (1973). Textural Features for Image Classification. IEEE Trans. Syst. Man, Cybern..

[118] Zhang R., Isola P., Efros A.A., Shechtman E., Wang O. The Unreasonable Effectiveness of Deep Features as a Perceptual Metric. Proceedings of the 2018 IEEE/CVF Conference on Computer Vision and Pattern Recognition.

[119] Heusel M., Ramsauer H., Unterthiner T., Nessler B., Hochreiter S., Guyon I., Luxburg U.V., Bengio S., Wallach H., Fergus R., Vishwanathan S., Garnett R. (2017). GANs Trained by a Two Time-Scale Update Rule Converge to a Local Nash Equilibrium. Advances in Neural Information Processing Systems.

[120] Krizhevsky A., Sutskever I., Hinton G.E., Pereira F., Burges C., Bottou L., Weinberger K. (2012). ImageNet Classification with Deep Convolutional Neural Networks. Advances in Neural Information Processing Systems.

[121] Hendrycks D., Dietterich T. (2019). Benchmarking Neural Network Robustness to Common Corruptions and Perturbations. arXiv.

[122] Torralba A., Efros A.A. Unbiased look at dataset bias. Proceedings of the CVPR 2011.

[123] Ronneberger O., Fischer P., Brox T., Navab N., Hornegger J., Wells W.M., Frangi A.F. U-Net: Convolutional Networks for Biomedical Image Segmentation. Proceedings of the Medical Image Computing and Computer-Assisted Intervention—MICCAI 2015, Munich, Germany, 5–9 October 2015.

[124] He K., Zhang X., Ren S., Sun J. Deep Residual Learning for Image Recognition. Proceedings of the 2016 IEEE Conference on Computer Vision and Pattern Recognition (CVPR).

[125] Loshchilov I., Hutter F. Decoupled Weight Decay Regularization. Proceedings of the International Conference on Learning Representations.

